# Target-tracking control method for autonomous vehicles based on hyperbolic-tangent line-of-sight guidance and odometry error compensation

**DOI:** 10.1371/journal.pone.0322648

**Published:** 2025-05-19

**Authors:** Xiaosong Liu, Huanhai Zhu, Zebiao Shan, Qingsong Lu, Liben He

**Affiliations:** 1 School of Electronic and Information Engineering, Changchun University of Science and Technology, Changchun, Jilin, China; 2 Joint School-Enterprise Technological Innovation Laboratory of Jilin Province for Intelligent Composite Robot, Changchun University of Science and Technology, Changchun, Jilin, China; National University of Singapore, SINGAPORE

## Abstract

The target-tracking accuracy of autonomous vehicles is closely related to that of onboard sensors. Methods such as image processing and base station positioning are susceptible to various types of interference in real-world scenarios, resulting in sensor data errors or even losses that ultimately affect the tracking accuracy of autonomous vehicles. This study proposes a target-tracking control method that relies solely on wheel odometry to address this issue. This method incorporates an extended state observer to compensate for the cumulative errors generated by the odometry mechanism, effectively enhancing the robustness and accuracy of the system in complex environments. In addition, a hyperbolic-tangent line-of-sight guidance strategy based on a partition-switching mechanism is designed to improve the dynamic response capability of an autonomous vehicle. This strategy nonlinearly adjusts the tracking error to generate the desired heading angle and velocity, ensuring that the target path tracking is rapid and smooth. First, we establish a mathematical model of an autonomous vehicle and combine the hyperbolic-tangent line-of-sight guidance strategy with a noise-resistant active disturbance rejection controller to achieve high-precision target tracking in dynamic environments. Second, an extended state observer is employed to perform real-time observations and compensate for unknown disturbances during localization, significantly reducing the impact of cumulative errors. Finally, the effectiveness of the proposed method is validated using numerical simulations and real vehicle experiments. The experimental results demonstrate that, compared with the ET-Fuzzy-MPC method, the proposed method lowered the average position tracking error by 45.39% under complex road conditions. In practical curved-path tests, the vehicle's tracking error remained stable to within 0.192 m, representing a significant improvement in the target tracking accuracy and dynamic response performance.

## 1. Introduction

Differentially driven autonomous vehicles find wide application in geological exploration, emergency rescue, and warehouse logistics because of their simple structure, high maneuverability, and strong adaptability [1–5]. Autonomous vehicle platforms integrate advanced hardware and software technologies that can perceive and understand the surroundings of a vehicle, thereby enabling them to make autonomous decisions and execute appropriate actions. Accurate target tracking is a core element of efficient navigation in autonomous vehicles because it ensures that targets can be tracked accurately and quickly in complex and dynamic environments. Therefore, target-tracking technology has become a key research focus, with experts in various fields continuously exploring new methods and algorithms to improve the localization accuracy and tracking performance of autonomous vehicles under different application scenarios.

Researchers have proposed various guidance strategies to control target tracking and address its associated challenges in complex environments. The core guidance strategy involves adjusting the robot's trajectory to track the movement of the target. The mainstream guidance strategies include pure pursuit, Stanley, and line-of-sight (LOS). The pure pursuit strategy maintains a specific geometric relationship between the robot and target and is suitable for tracking smooth paths. For example, to improve the tracking accuracy of low-speed autonomous vehicles on straight and curved paths, [6] proposed an improved pure pursuit strategy based on Ackerman steering. In addition, reference [7] proposed a tracking control method based on an improved pure pursuit strategy that dynamically adjusts the look-ahead distance to adapt to different road scenarios. The Stanley strategy calculates the steering angle by minimizing the lateral and heading errors to stabilize the tracking. In [8], predictive components were added to the Stanley strategy, enabling future states to be predicted and adjusted. This method calculates the steering angle by combining the output of the Stanley strategy with additional predictive terms, improving the tracking stability and accuracy. An improved Stanley control strategy (IMP-ST) to enhance the tracking control performance was proposed in [9]. Compared to the two guidance methods mentioned above, the LOS strategy is more widely used in tracking control systems of autonomous vehicles and ships because of its simplicity, efficiency, strong anti-interference performance, and ease of implementation. It also possesses rapid path correction capabilities, rendering it particularly suitable for dynamic scenarios [10]. In [11], an LOS guidance strategy based on finite-time drift angles to address the tracking issues of unmanned ships under dynamic disturbances was proposed. A foresight angle into the traditional LOS strategy to compensate for the output LOS angle was introduced in [12].

Although guidance strategies perform well in path correction, they cannot guarantee robustness and control accuracy in complex environments. Particularly, when external disturbances, system uncertainties, or complex paths are involved, guidance algorithms cannot fully adapt to trajectory deviations and dynamic adjustments. Therefore, it is necessary to optimize guidance strategies to increase their suitability for complex paths and use control algorithms to provide effective feedback compensation, which can enhance the system's robustness and disturbance rejection capability. Various control algorithms have been proposed to enhance the target tracking capability of autonomous vehicles, including sliding mode control, vision-based control, receding horizon control, and neural networks. In [13], a nonlinear model predictive control (MPC) algorithm was used to effectively localize omnidirectional mobile robots in dynamic environments. Furthermore, reference [14] designed a robust control scheme based on disturbance observers and integral sliding mode control, enabling robot systems to asymptotically track the desired speeds while mitigating compound disturbances. Reference [15] proposed a method that combines the Stanley algorithm with proportional-integral-derivative (PID) control to further enhance the tracking capability of autonomous vehicles. Reference [16] addressed the complex constraints in tracking autonomous logistics vehicles by proposing an event-triggered and adaptive optimization-based MPC method, integrating disturbance observers with fuzzy control to optimize the parameters and improve the path tracking smoothness and accuracy. Reference [17] adopted an approach that combined proportional-integral (PI) control and pure pursuit control for target trajectories with significant curvature variations, effectively reducing tracking errors. Reference [18] proposed an optimized LOS guidance strategy based on sliding mode control for tracking problems in snake robots, effectively reducing the target tracking deviation errors. Reference [19] investigated a strategy that combined adaptive LOS and MPC to enhance the guidance performance via real-time adjustment of the look-ahead distance parameter. Reference [20] introduced a novel neural network that employs integral sliding mode control methods and combines certain compensation strategies to minimize the road impact on vehicles. The simulation results indicated that this adaptive neural network controller can effectively track preplanned paths. To address system uncertainties, nonlinearities, and external disturbances affecting trajectory control, reference [21] proposed a vehicle trajectory tracking system with dynamic errors and designed an active disturbance rejection controller (ADRC) for trajectory tracking based on performance indicators and disturbance compensation. Lee et al. designed a linear quadratic optimal preview controller for tracking desired paths, achieving high control accuracy for various driving environments [22]. CERON et al. investigated the problem of lateral control in vehicles, proposing a robust controller using μ-synthesis based on steering wheel angle inputs while accounting for model uncertainties [23]. Reference [24] applied linear active disturbance rejection control (LADRC) to the steering control of six-wheel autonomous vehicles to address lateral and longitudinal slip issues at the wheel-terrain contact surface. In the abovementioned methods, noise and uncertainty often adversely affect the system's performance. Sensor noise, environmental disturbances, and external uncertainties may render traditional control methods insufficient for providing adequate accuracy and stability in practical applications. To address this, [25] proposed a linear programming-based signal differentiator for signal processing in high-noise environments, providing optimal worst-case accuracy.

Although the aforementioned research methods have improved target tracking accuracy to a certain extent, they still have shortcomings. First, these methods generally involve various controller parameters, which hinders parameter tuning. Many control algorithms rely on numerous parameters, and adjusting these parameters requires substantial time and expertise, making the prompt deployment of the algorithms in practical applications more challenging. Second, these methods typically exhibit high computational complexity and require high-performance hardware to support their operations, which significantly increases the system costs, rendering these methods unsuitable for low-cost application scenarios. Finally, most existing methods assume that the environment perception systems of autonomous vehicles are reliable, ignoring the impact of sensor noise, uncertainties, and environmental changes on the vehicle's target tracking performance. This degrades the robustness in complex environments. Moreover, odometry errors are a significant factor affecting the accuracy of target tracking. Owing to limitations such as friction, terrain undulations, and sensor accuracy, odometry errors accumulate during prolonged operations. This accumulation eventually causes the robot to deviate from its trajectory when executing LOS guidance strategies, severely affecting the overall trajectory tracking performance.

This study proposes a hyperbolic-tangent nonlinear adjustment-based LOS guidance strategy and a target tracking control method that relies solely on wheel odometry and that employs an extended state observer (ESO) to provide real-time compensation for cumulative errors to overcome the challenges posed by cumulative odometry errors to the target tracking accuracy of autonomous vehicles in complex dynamic environments; this serves to enhance the reliability, accuracy, and dynamic adaptability of autonomous vehicle target tracking systems and promotes their widespread application in areas such as unstructured terrain navigation, warehousing and logistics, and urban traffic. This strategy nonlinearly adjusts the error between the autonomous vehicle and the target, generating the desired heading angle and velocity. In addition, it exhibits excellent dynamic response and target-tracking accuracy in scenarios with rapidly changing paths and complex environments. A wheel odometry localization model is established to address the accuracy degradation caused by cumulative errors in wheel odometry localization. After analyzing disturbances during the localization process, an ESO is employed to observe and reconstruct the states of unknown nonlinear systems in real time. A disturbance compensator is then utilized to effectively compensate for the cumulative errors, thereby enhancing the robustness and accuracy of the system. Finally, numerical simulations and real vehicle experiments are conducted to validate the proposed method.

The remainder of this paper is organized as follows. Section 2 defines the problem, including the mathematical model of the autonomous vehicle and the error dynamic model used for target tracking. Section 3 introduces the odometry-based localization algorithm, which includes the localization process, odometry compensation algorithm, and stability analysis. Section 4 introduces the design of both the traditional and hyperbolic-tangent LOS guidance strategies. Section 5 describes the design of the active disturbance rejection control (ADRC) algorithm under noise interference. Section 6 presents the numerical simulations and real-world road tests used to validate the proposed method. Section 7 summarizes the main conclusions and discusses future research directions.

## 2. Problem description

### 2.1. Mathematical model of the autonomous vehicle

**Fig 1:**
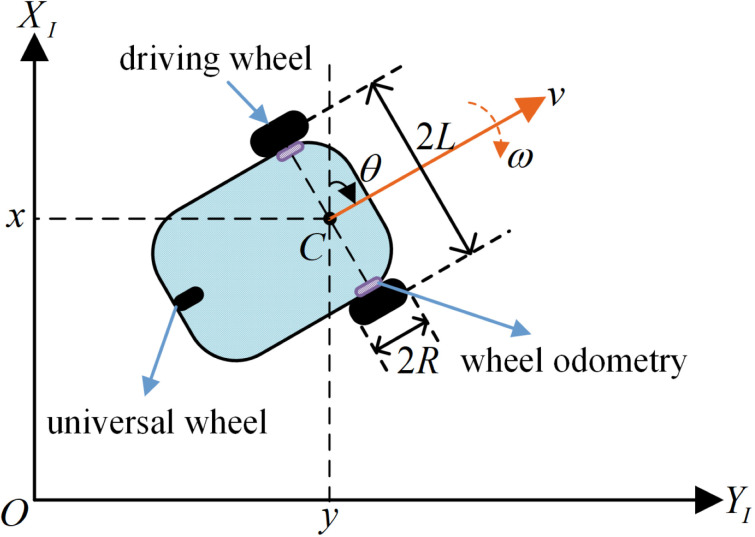
Autonomous vehicle model.

The structure of a differential-drive autonomous vehicle is illustrated in Fig 1. The two front wheels are driving wheels powered by an independently driven direct current (DC) motor. The left and right driving wheels are equipped with an odometer for vehicle speed measurements and recording the distance traveled. The rear wheel is a universal wheel that only provides support.

A global coordinate system $\begin{array}{c} \left\{X_{I},Y_{I}\right\} \end{array}$ is defined relative to the ground to describe the motion of the autonomous vehicle. The mathematical model of the autonomous vehicle incorporates a kinematic model and a dynamic model. The wheels are assumed to experience only rolling friction (not sliding friction) with the road surface. The pose of the mobile robot is defined as $\begin{array}{c} q={\left[xy\theta \right]}^{T} \end{array}$, where $\begin{array}{c} x \end{array}$ and $\begin{array}{c} y \end{array}$ represent the vehicle's coordinates in the global coordinate system $\begin{array}{c} \left\{X_{I},Y_{I}\right\} \end{array}$, and $\begin{array}{c} \theta \end{array}$ represents the heading angle [18]. Therefore, the mathematical model of the autonomous vehicle is


$$\dot{q}=\Omega (qxi,$$ (1)

where $\begin{array}{c} \dot{q}={\left[\dot{x}\dot{y}\omega \right]}^{T} \end{array}$ represents the derivative of $\begin{array}{c} q \end{array}$, $\begin{array}{c} \Omega (q)=\left[\begin{array}{cc} \cos\theta & 0\\ \sin\theta & 0\\ 0 & 1 \end{array}\right] \end{array}$, and $\xi ={\left[v\omega \right]}^{\text{T}}$, where $\begin{array}{c} v \end{array}$ and $\begin{array}{c} \omega \end{array}$ denote the linear and angular velocities of the vehicle, respectively.

The dynamic model of the autonomous vehicle is described as follows. According to the torque balance principle, the acceleration torque of the vehicle equals the difference between the driving torques of the two driving wheels [23]:


$$I_{v}\ddot{\theta }=F_{l}L-F_{r}L.$$ (2)

According to Newton’s second law of motion, we have


$$M\dot{v}=F_{r}+F_{l}.$$ (3)

The dynamic characteristics of the vehicle's left and right wheels are


$$I_{w}{\ddot{\theta }}_{r}+c{\dot{\theta }}_{r}=ku_{r}-rF_{r},$$ (4)

$$I_{w}{\ddot{\theta }}_{l}+c{\dot{\theta }}_{l}=ku_{l}-rF_{l},$$ (5)

where the subscripts l and r represent the left and right wheels, respectively; c represents the rolling friction coefficient between the driving wheels and road surface; and k denotes the transmission gain between the driving motor and reduction gears and is given by $k=nk_{m}/R_{a}\;$, where n is the gear reduction ratio, $k_{m}$ is the electromagnetic torque constant of the motor, and $R_{a}$ is the motor armature resistance. The kinetic parameters are shown in Table 1.

**Table 1:** Dynamic parameters.

Physical Meaning	Symbol	Unit	Value
Mass of the autonomous vehicle	M	kg	50
Moment of inertia about the center of gravity of the autonomous vehicle	$I_{v}$	$\text{kg}\cdot {\text{m}}^{\text{2}}$	10
Moment of inertia of the wheels	$I_{w}$	$\text{kg}\cdot {\text{m}}^{\text{2}}$	0.05
Half the width of the autonomous vehicle	L	m	0.3
Radius of the wheels	R	m	0.2
Friction coefficient between the tires and the ground	c	$\text{kg}\cdot {\text{m}}^{\text{2}}/s$	0.15
Motor and transmission mechanism drive gain	k	Nm/V	20

Based on the principle of the motion of a differential-drive autonomous vehicle, the linear and angular velocities of the vehicle are given by:


$$v=\frac{v_{r}+v_{l}}{2},$$ (6)

$$\omega =\frac{v_{l}-v_{r}}{2L}.$$ (7)

Combining Equations (2)–(7) gives the rates of change of the vehicle's linear and angular velocities as


$$\dot{v}=-\frac{2c}{MR^{2}+2I_{w}}v+\frac{kR}{MR^{2}+2I_{w}}\left(u_{r}+u_{l}\right),$$ (8)

$$\dot{\omega }=-\frac{2cL^{2}}{I_{v}R^{2}+2I_{w}L^{2}}\dot{\phi }+\frac{kRL}{I_{v}R^{2}+2I_{w}L^{2}}\left(u_{l}-u_{r}\right),$$ (9)

respectively. During actual operations, factors such as backlash in the motor gearbox, body oscillations, and instability of the center of gravity can cause disturbances in the vehicle body. The total disturbance of the vehicle caused by external factors is defined as $D={\left[\begin{array}{cc} D_{v} & D_{\omega } \end{array}\right]}^{\text{T}}$, where $D_{v}$ and $D_{\omega }$ represent the disturbances to the vehicle's linear and angular velocities, respectively. The dynamic model of the autonomous vehicle can then be expressed as


$$\dot{\xi }=A\xi +Bu+D,$$ (10)

where $A=\left[\begin{array}{cc} a_{1} & 0\\ 0 & a_{2} \end{array}\right]$, $B=\left[\begin{array}{cc} b_{1} & b_{1}\\ b_{2} & -b_{2} \end{array}\right]$, $\begin{array}{c} u=\left[\begin{array}{c} u_{l}\\ u_{r} \end{array}\right] \end{array}$, $a_{1}=-\frac{2c}{MR^{2}+2I_{w}}$, $a_{2}=-\frac{2cL^{2}}{I_{v}R^{2}+2I_{w}L^{2}}$, $b_{1}=\frac{kR}{MR^{2}+2I_{w}}$, and $b_{2}=\frac{kRL}{I_{v}R^{2}+2I_{w}L^{2}}$.

### 2.2. Error dynamics model for vehicle target tracking

**Fig 2:**
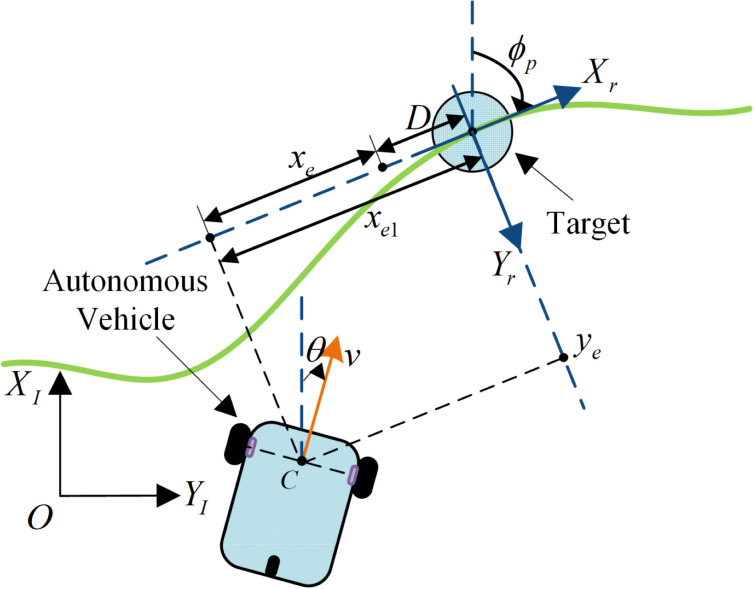
Plot of the target tracking error of the autonomous vehicle.


The target tracking error is illustrated in Fig 2. A path tangential coordinate system, $\left\{X_{r},Y_{r}\right\}$, is established to facilitate the description of the position and motion relationship between the autonomous vehicle and the target. This coordinate system is rotated relative to the global coordinate system by the angle $\phi_{p}$, which is the heading angle of the target. The position coordinates of the target in the global coordinate system are defined as $x_{r}$ and $y_{r}$, and the angle $\phi_{p}$ satisfies the following:

$$\phi_{p}=\mathrm{atan}2({\dot{y}}_{r}(t),{\dot{x}}_{r}(t)).$$ (11)

To further describe the position deviation between the autonomous vehicle and the target, the position error is defined as $q_{e1}={\left[\begin{array}{ccc} x_{e\text{1}} & y_{e} & \theta_{e} \end{array}\right]}^{\text{T}}$, where $x_{e\text{1}}$ represents the longitudinal distance between the target and autonomous vehicle, $y_{e}$ denotes the lateral error, and $\theta_{e}$ denotes the angular error. The position error between the target $\left(x_{r},y_{r},\phi_{p}\right)$ and autonomous vehicle (x,y,θ) is therefore


$$\left[\begin{array}{c} {}*35lx_{e\text{1}}\\ \begin{array}{c} y_{e}\\ \theta_{e} \end{array} \end{array}\right]={\left[\begin{array}{ccc} \cos\phi_{p} & -\sin\phi_{p} & 0\\ \sin\phi_{p} & \cos\phi_{p} & 0\\ 0 & 0 & 1 \end{array}\right]}^{\text{T}}\left[\begin{array}{cc} & \begin{array}{c} x-x_{r}\\ y-y_{r} \end{array}\\ & \theta -\phi_{p} \end{array}\right].$$ (12)

During target tracking, the autonomous vehicle is required to remain a distance D behind the target. Therefore, the target tracking error is defined as $q_{e}={\left[\begin{array}{ccc} x_{e} & y_{e} & \theta_{e} \end{array}\right]}^{\text{T}}$. Fig 2 reveals that the longitudinal error during tracking is $x_{e}=x_{e1}-D$. Based on the mathematical model of the autonomous vehicle, which is given in Equation (1), we obtain


$$\left\{ \begin{array}{c} @l{\dot{x}}_{e}=v\cos(\theta -\phi_{p})+{\dot{\phi }}_{p}y_{e}-v_{r}\\ {\dot{y}}_{e}=v\sin(\theta -\phi_{p})-{\dot{\phi }}_{p}x_{e}\\ {\dot{\theta }}_{e}=\dot{\theta }-{\dot{\phi }}_{p} \end{array}\right.,$$ (13)

where $v_{r}$ denotes the linear velocity of the reference trajectory. According to the analysis above, the target-tracking task of the autonomous vehicle is to find a bounded input τ, such that $q_{e}={\left[\begin{array}{ccc} x_{e} & y_{e} & \theta_{e} \end{array}\right]}^{\text{T}}$ converges to zero as $\underset{t\to \infty }{\lim}\;\left\|q_{e}\right\|=0$, regardless of the initial error.

## 3. Odometry-based localization algorithm

### 3.1. Localization process based on odometry

Wheel odometers are commonly used vehicle sensors that measure v and heading angle θ. The vehicle localization process based on odometry is incremental. Assuming that the vehicle's position at time t is $X_{t}$ and that the movement increment during the time interval from t to t+1 is $\Delta X_{t}^{t+1}$, the vehicle's position at time t+1 can be obtained. This localization process can be described by


$$X_{t+1}=X_{t}+\Delta X_{t}^{t+1},$$ (14)

where $X_{t}=[x_{t},y_{t}]$ represents the vehicle's coordinates in the global coordinate system $\left\{X_{\text{I}},Y_{\text{I}}\right\}$ at time t; $\Delta X_{t}^{t+1}={\left[\Delta x\Delta y\right]}^{\text{T}}=T\cdot L(\theta )$ represents the movement increment of the vehicle during the time interval from t to t+1; Δx and Δy represent the movement increments of the vehicle in the *X* and *Y* directions during the time interval from t to t+1, respectively; R represents the radius of the wheels; L(θ) represents the distance traveled along the direction of the vehicle's heading angle; and $T={\left[\cos\theta \sin\theta \right]}^{\text{T}}$ represents the projection matrix that projects $\Delta X_{t}^{t+1}$ onto the *X* and *Y* directions.

A deviation exists between the actual coordinates of the vehicle and those calculated using the odometry because of various uncertainties. The causes of odometry measurement deviations are complex and coupled. However, they can generally be categorized into four types: measurement errors in the system caused by the sensor's intrinsic accuracy ($\tilde{\theta }$ and $\tilde{v}$), uncertain disturbances (D(t)) caused by friction between the ground and wheels as well as the vehicle's load, modeling errors ($E(\tilde{\theta },\tilde{v})$) caused by imperfections in the established system model, and errors ($F(\tilde{\theta },\tilde{v})$) caused by the linearization of the nonlinear system. Considering the above factors, the odometry localization model can be described as


$$\begin{array}{c} \begin{array}{cc} & \;X_{t+1}=X_{t}+\Delta X_{t}^{t+1}\\ & \;=X_{t}+\Delta X(o)+D(t)+E(\tilde{\theta },\tilde{v})+F(\tilde{\theta },\tilde{v}), \end{array} \end{array}$$ (15)

where ΔX(o) represents the actual movement increment of the vehicle.

### 3.2. Odometry compensation algorithm

Traditional localization methods reduce the impact of disturbances by establishing models and combining multiple measurements. However, system models are difficult to establish accurately, and system parameters vary with environmental changes. In addition, because the localization process is incremental, errors in historical odometry measurements accumulate over time, leading to increased localization errors.

In contrast to traditional methods, the proposed method applies the concept of ADRC to the odometry localization compensation. The analysis above indicates that errors in the odometry measurements lead to higher trajectory errors. In this study, a disturbance compensation strategy is proposed. In the proposed compensation strategy, all factors causing errors are unified and treated as one disturbance denoted as $f_{all}$. Setting $X={\left[xy\right]}^{\text{T}}$ and $\begin{array}{c} T\cdot v=\Delta \dot{X}(o) \end{array}$, Equation (15) can be rewritten as


$$\dot{X}=T\cdot v+f_{all},$$ (16)

where $\begin{array}{c} \dot{X} \end{array}$ represents the derivative of the localization coordinate X, and $f_{all}=D(t)+E(\tilde{\theta },\tilde{v})+F(\tilde{\theta },\tilde{v})$. The quantity T·v represents the velocity components of the vehicle in the *X* and *Y* directions.

In ADRC, an ESO is used to observe disturbances in the system. The higher the order of the ESO, the stronger its ability to observe rapidly changing disturbances; however, it also requires a longer computation time. In vehicle target tracking, the computational accuracy and speed must be balanced. The derivative of the total disturbance $f_{all}$ in the odometry localization process changes slowly and is almost zero. Therefore, a second-order ESO is selected and can be expressed as


$$\left\{ \begin{array}{c} @l\varepsilon_{1}=s_{1}-X\\ {\dot{s}}_{1}=s_{2}+T\cdot v-\beta_{1}\varepsilon_{1}\\ {\dot{s}}_{2}=s_{3}-\beta_{2}\varepsilon_{1}\\ {\dot{s}}_{3}=-\beta_{3}\varepsilon_{1} \end{array}\right.,$$ (17)

where $\begin{array}{c} s_{1} \end{array}$ is the observed value of X, $s_{2}$ is the observed value of $f_{all}$, $s_{3}$ is the observed value of $\begin{array}{c} {\dot{f}}_{all} \end{array}$, $\begin{array}{c} \varepsilon_{1} \end{array}$ represents the error between the observed and actual values, and $\beta_{1}$, $\beta_{2}$, and $\beta_{3}$ are the gain parameters of the ESO.

The compensated coordinates are given as


$$\dot{X}=T\cdot v+f_{all}-s_{2}.$$ (18)

### 3.3. Stability analysis

**Assumption 1:** Odometry disturbances change slowly:


$${\ddot{f}}_{all}=0.$$ (19)

**Theorem 1**: Equation (17) is used to observe disturbances in the odometry localization process. If the disturbance $f_{all}$ satisfies Assumption 1 and the parameters of the ESO ensure that $A=\left[\begin{array}{ccc} -\beta_{1} & 1 & 0\\ -\beta_{2} & 0 & 1\\ -\beta_{3} & 0 & 0 \end{array}\right]$ is a Hurwitz matrix, then the observation error $\varepsilon ={\left[\varepsilon_{1},\varepsilon_{2},\varepsilon_{3}\right]}^{\text{T}}$ is asymptotically stable.

**Proof**: The observation error is defined as


$$\left\{ \begin{array}{c} @l\varepsilon_{1}=s_{1}-X\\ \varepsilon_{2}=s_{2}-f_{all}\\ \varepsilon_{3}=s_{3}-{\dot{f}}_{all} \end{array}\right..$$ (20)

Taking the derivative of Equation (19) and substituting into Equations (16) and (17), we obtain


$$\left\{ \begin{array}{c} @l{\dot{\varepsilon }}_{1}=-\beta_{1}\varepsilon_{1}+\varepsilon_{2}\\ {\dot{\varepsilon }}_{2}=-\beta_{2}\varepsilon_{1}+\varepsilon_{3}\\ {\dot{\varepsilon }}_{3}=-\beta_{3}\varepsilon_{1}+{\ddot{f}}_{all} \end{array}\right..$$ (21)

From Assumption 1, ${\ddot{f}}_{all}=0$; thus, Equation (21) can be rewritten as:


$$\dot{\varepsilon }=A\varepsilon .$$ (22)

A is a Hurwitz matrix; thus, a matrix P satisfies:


$$A^{\text{T}}P+PA=-I.$$ (23)

Choosing the Lyapunov function as:


$$V(\varepsilon )=\varepsilon^{\text{T}}P\varepsilon ,$$ (24)

and taking the derivative of this function, we have


$$\mathrm{\backslash [}\begin{array}{cc} & \;\dot{V}(\varepsilon )={\dot{\varepsilon }}^{\text{T}}P\varepsilon +\varepsilon^{\text{T}}P^{\text{T}}\dot{\varepsilon }\\ & =\varepsilon^{\text{T}}A^{\text{T}}P\varepsilon +\varepsilon^{\text{T}}PA\varepsilon \\ & =\varepsilon^{\text{T}}\left(A^{\text{T}}P+PA\right)\varepsilon \\ & =-{\left\|\varepsilon \right\|}^{2}. \end{array}\mathrm{\backslash ]}$$ (25)

When $\begin{array}{c} \varepsilon \to 0 \end{array}$, the observation error in Equation, $\varepsilon ={\left[\varepsilon_{1},\varepsilon_{2},\varepsilon_{3}\right]}^{\text{T}}$, is asymptotically stable.

## 4. Design of hyperbolic tangent line-of-sight guidance strategy

### 4.1. Traditional line-of-sight guidance strategy

**Fig 3:**
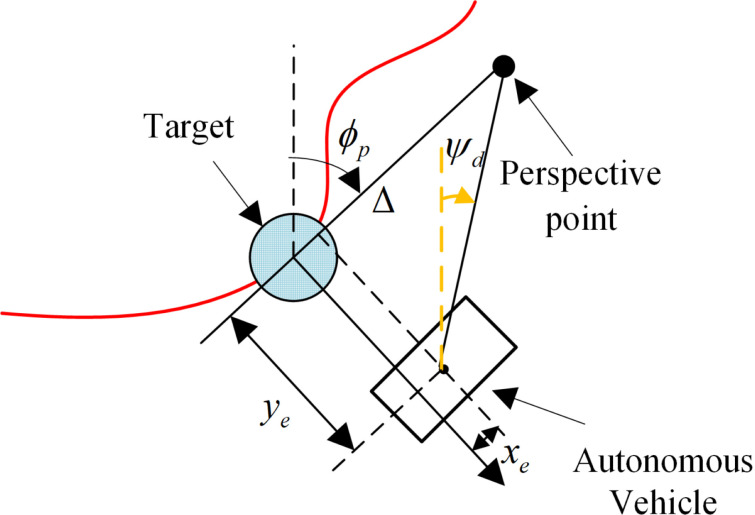
Diagram of the traditional LOS guidance strategy.

The traditional LOS guidance strategy, which is shown in Fig 3, is based on adjusting the vehicle's heading angle to always aim at a look-ahead point on the target path, thereby achieving target tracking. In the traditional LOS strategy, the look-ahead distance is a key parameter for controlling the vehicle, and it directly affects the tracking accuracy and response speed.

The traditional LOS guidance strategy is expressed as [26]:


$$\psi_{d}≜\phi_{p}-\arctan(\frac{y_{e}}{\Delta }),\Delta >0,$$ (26)

where $\psi_{d}$ is the desired heading angle, $\phi_{p}$ is the target heading angle, $y_{e}$ is the lateral error, and Δ is the look-ahead distance. When the autonomous vehicle's actual heading angle θ tracks the desired heading angle $\psi_{d}$ and maintains a nonzero forward speed, it gradually converges to the reference trajectory.

### 4.2. Hyperbolic tangent line-of-sight guidance strategy

A nonlinear LOS guidance strategy based on the hyperbolic tangent function ($\tanh(x)=\frac{{\text{e}}^{x}-{\text{e}}^{-x}}{{\text{e}}^{x}+{\text{e}}^{-x}}$) is proposed to overcome the limitations of the traditional LOS strategy and enhance the rapid target tracking performance of autonomous vehicles. For small inputs, this function is approximately linear; for large inputs, the output is limited to the range [-1, 1]. Consequently, tanh(x) avoids excessive correction for large errors while providing precise linear control for small errors. Compared with the linear proportional control of the traditional LOS strategy, the tanh(x) function adaptively adjusts the error responses, balancing the control requirements for both large and small errors. Therefore, in this study, a tanh(x) function is introduced into the traditional LOS strategy to perform nonlinear error adjustments.

#### 4.2.1. Design of the heading guidance.

The adjustment of the heading angle of the autonomous vehicle relies primarily on feedback from the lateral error $y_{e}$. A nonlinear adjustment is applied to the lateral error $y_{e}$ using the tanh(x) function, and a partition-switching mechanism $\vartheta_{y}$ is introduced for error correction to avoid aggressive control caused by large errors in the traditional LOS strategy.

The heading guidance law in the hyperbolic LOS (hereafter, “HLOS”) guidance strategy is


$$\psi_{d}=\phi_{p}-\arctan(k_{y}\tanh(a_{y}y_{e})+\vartheta_{y}),$$ (27)

where $\psi_{d}$ is the desired heading angle, $\phi_{p}$ is the heading angle from the reference trajectory, $y_{e}$ is the lateral error, $k_{y}>0$ and $a_{y}>0$ are the control gain parameters used to adjust the system's response to lateral errors, and $\vartheta_{y}$ is a partition-switching mechanism that adjusts and compensates for the error feedback.

The heading guidance law in Equation (27) applies a nonlinear adjustment to the lateral deviation using the function $\tanh\left(a_{y}y_{e}\right)$. When $y_{e}$ is small, $\tanh(a_{y}y_{e}approxa_{y}y_{e}$, which provides sensitive adjustments to $y_{e}$, allowing the autonomous vehicle to quickly track the target. When $y_{e}$ is large, $\tanh\left(a_{y}y_{e}\right)$ saturates, which prevents excessive responses from the autonomous vehicle.

#### 4.2.2. Velocity guidance.

Speed control is as crucial as heading angle control in complex target-tracking tasks. The speed control law adjusts the robot's speed based on the target's speed $v_{r}$ and longitudinal error $x_{e}$, ensuring the target is smoothly tracked. In addition, the tanh(x) function is applied to the longitudinal error feedback for nonlinear adjustment, ensuring smooth speed adjustments under varying error conditions.

The velocity guidance law in the HLOS strategy is expressed as


$$v_{d}=\frac{v_{r}-k_{x}\tanh(a_{x}x_{e})-\vartheta_{x}}{\cos(\theta -\phi_{p})},$$ (28)

where $v_{d}$ is the desired speed, $k_{x}>0$ and $a_{x}>0$ are control gain parameters that adjust the system's response to the longitudinal error, and $\vartheta_{x}$ is a partition-switching mechanism. In Equation (28), $\tanh\left(a_{x}x_{e}\right)$ applies a nonlinear adjustment to $x_{e}$. When $x_{e}$ is large, the speed is limited to avoid instability caused by excessive control signals. When $x_{e}$ is small, the speed response is rapid, drawing it closer to the target speed to stabilize the tracking.

#### 4.2.3. Design of the partition-switching mechanism.

In the HLOS strategy, to ensure smooth control responses from the autonomous vehicle across different error ranges and avoid fluctuations in the control signals, the partition-switching mechanisms $\vartheta_{y}$ and $\vartheta_{x}$ are designed to further compensate for lateral and longitudinal errors.

For the heading angle control, the partition-switching mechanism $\vartheta_{y}$ smooths the feedback intensity for lateral errors. Adjusting the nonlinearity of the lateral error feedback ensures that adjustments to the heading angle are controlled and smooth under varying error conditions. The partition-switching mechanism $\vartheta_{y}$ is divided into two actions based on the magnitude of the lateral error: nonlinear forced corrections for large errors and smooth transitions for small errors. The partition-switching mechanism $\vartheta_{y}$ is expressed as


$${\dot{\vartheta }}_{y}=\left\{ \begin{array}{cc} ll-k_{\vartheta_{y}}sgn(\vartheta_{y}) & \\ +\frac{v_{d}y_{e}}{\sqrt{1+{\left(k_{y}\tanh(a_{y}y_{e})+\vartheta_{y}\right)}^{2}}} & \\ -\frac{k_{y}v_{d}(\left|y_{e}\right|-y_{e}\tanh(a_{y}y_{e}))}{\vartheta_{y}\sqrt{1+{\left(k_{y}\tanh(a_{y}y_{e})+\vartheta_{y}\right)}^{2}}}, & \left|\vartheta_{y}\right|\geq \epsilon_{\vartheta_{y}},\\ -k_{\vartheta_{y}}sgn(\vartheta_{y}) & \\ +\frac{v_{d}y_{e}}{\sqrt{1+{\left(k_{y}\tanh(a_{y}y_{e})+\vartheta_{y}\right)}^{2}}}, & \left|\vartheta_{y}\right|<\epsilon_{\vartheta_{y}} \end{array}\right.$$ (29)

where $k_{\vartheta_{y}}$ is the adjustment parameter, $\epsilon_{\vartheta_{y}}$ is the critical point for error switching (which determines the timing of the partition-switching mechanism), and *sgn* is the sign function that indicates the direction of the error.

The partition-switching mechanism $\vartheta_{x}$ adjusts the feedback for longitudinal errors to ensure that the speed control is smooth, and it is given as


$${\dot{\vartheta }}_{x}=\left\{ \begin{array}{cc} ll-k_{\vartheta_{x}}sgn(\vartheta_{x})+x_{e} & \\ -\frac{k_{x}(\left|x_{e}\right|-x_{e}\tanh(a_{x}x_{e}))}{\vartheta_{x}}, & \left|\vartheta_{x}\right|\geq \epsilon_{\vartheta_{x}},\\ -k_{\vartheta_{x}}sgn(\vartheta_{x})+x_{e}, & \left|\vartheta_{x}\right|<\epsilon_{\vartheta_{x}} \end{array}\right.$$ (30)

where $k_{\vartheta_{x}}$ is the adjustment parameter, $\epsilon_{\vartheta_{x}}$ is the critical point for error switching, and \sgn(·) is the sign function.

The partition-switching mechanisms $\vartheta_{x}$ and $\vartheta_{y}$ perform error partition control using the switching points. When the thresholds $\epsilon_{\vartheta_{x}}$ and $\epsilon_{\vartheta_{y}}$ are exceeded, a nonlinear adjustment mechanism is applied to desensitize the autonomous vehicle to large errors; when these thresholds are not exceeded, small errors are not neglected.

**Theorem 2**: If the tracking errors $\psi_{e}=\theta -\psi_{d}$ and $v_{e}=v-v_{d}$ are sufficiently small, the target tracking errors $x_{e}$ and $y_{e}$ and the partition-switching mechanisms $\vartheta_{x}$ and $\vartheta_{y}$ can achieve asymptotic stability via the HLOS algorithm expressed in Equations (27)–(30).

**Proof**:

From Equation (27), we have


$$\sin(\psi_{d}-\phi_{p})=-\frac{k_{y}\tanh(a_{y}y_{e})+\vartheta_{y}}{\sqrt{1+{\left(k_{y}\tanh(a_{y}y_{e})+\vartheta_{y}\right)}^{2}}},$$ (31)

$$\cos(\psi_{d}-\phi_{p})=\frac{1}{\sqrt{1+{\left(k_{y}\tanh(a_{y}y_{e})+\vartheta_{y}\right)}^{2}}},$$ (32)

According to Equations (27)–(30), the target tracking error dynamics can be expressed as:


$$\left\{ \begin{array}{c} @l{\dot{x}}_{e}\text{=}-k_{x}\tanh(a_{x}x_{e})+{\dot{\phi }}_{p}y_{e}-\vartheta_{x}\\ {\dot{y}}_{e}=-\frac{v_{d}(k_{y}\tanh(a_{x}x_{e})+\vartheta_{y})}{\sqrt{1+{\left(k_{y}\tanh(a_{x}x_{e})+\vartheta_{y}\right)}^{2}}}-{\dot{\phi }}_{p}x_{e}\\ \;+h_{\psi }\psi_{e}+h_{v}u_{e} \end{array}\right.,$$ (33)

where


$$\mathrm{\backslash [}\begin{array}{cc} & \#x0026;h_{\psi }=v_{d}\sin(\psi_{d}-\phi_{p})\frac{\cos\psi_{e}-1}{\psi_{e}},\\ & \#x0026;+v_{d}\cos(\psi_{d}-\phi_{p})\frac{\sin\psi_{e}}{\psi_{e}} \end{array}\mathrm{\backslash ]}$$ (34)

$$h_{v}=\sin(\theta -\phi_{p}).$$ (35)

The Lyapunov function can be constructed as


$$V_{1}=\frac{1}{2}(x_{e}^{2}+y_{e}^{2}+\varsigma^{\text{T}}\varsigma ).$$ (36)

When $\begin{array}{c} \varsigma \in {\;\;\;\Omega \;\;}_{1}:=\left\{\left|\vartheta_{x}\right|\geq \epsilon_{\vartheta_{x}},\left|\vartheta_{y}\right|\geq \epsilon_{\vartheta_{y}}\right\}, \end{array}$


$$\mathrm{\backslash [}\begin{array}{cc} & {\dot{V}}_{1}=x_{e}{\dot{x}}_{e}+y_{e}{\dot{y}}_{e}+\vartheta_{x}{\dot{\vartheta }}_{x}+\vartheta_{y}{\dot{\vartheta }}_{y}\\ & =-k_{x}\left|x_{e}\right|-k_{y}^{\prime }\left|y_{e}\right|-k_{\vartheta_{x}}\left|\vartheta_{x}\right|-k_{\vartheta_{y}}\left|\vartheta_{y}\right|\\ & +y_{e}(h_{\psi }\psi_{e}+h_{u}v_{e}), \end{array}\mathrm{\backslash ]}$$ (37)

Where $k_{y}^{\prime }=k_{y}v_{d}/\sqrt{1+{\left(k_{y}\tanh(a_{y}y_{e})+\vartheta_{y}\right)}^{2}}>0$. From Equations (34) and (35), we have the following:


$$\left|h_{\psi }\right|\leq v_{d},\left|h_{v}\right|\leq 1.$$ (38)

If the tracking errors $\psi_{e}$ and $v_{e}$ are sufficiently small, then


$${\dot{V}}_{1}\leq -k_{x}\left|x_{e}\right|-\kappa_{y}\left|y_{e}\right|-k_{\vartheta_{x}}\left|\vartheta_{x}\right|-k_{\vartheta_{y}}\left|\vartheta_{y}\right|\leq -k_{1}\sqrt{V_{1}},$$ (39)

where $\kappa_{y}={k^{\prime }}_{y}-(2v_{\max}+d\omega_{\max})\varepsilon_{\psi_{e}}-\varepsilon_{v_{e}}>0$ and $k_{1}=\sqrt{2}\min\left\{k_{x},\kappa_{y},k_{\vartheta_{x}},k_{\vartheta_{y}}\right\}$. Other cases can be similarly proven. In conclusion, $x_{e}$, $y_{e}$, $\vartheta_{x}$, and $\vartheta_{y}$ are asymptotically stable.

## 5. Design of an active disturbance rejection control algorithm

**Fig 4:**
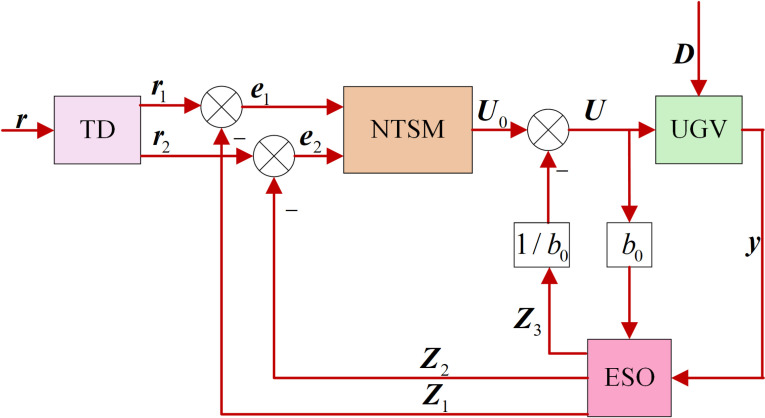
Block diagram of the active disturbance rejection controller.

Based on the HLOS strategy, this section describes the design of the ADRC heading angle and velocity controllers, which ensure the rapid convergence of the tracking errors $\psi_{e}=\theta -\psi_{d}$ and $v_{e}=v-v_{d}$, while estimating and compensating for disturbances using the ESO. The goal of the heading angle and velocity controllers is to enable the autonomous vehicle to accurately track the target in complex environments despite disturbances. The traditional ADRC, which is shown in Fig 4, consists of a tracking differentiator (TD), an ESO, and a nonlinear state error feedback (NLSEF) controller. The TD is primarily used to rapidly acquire input signals, including tracking and differential signals. The ESO estimates the state variables of the autonomous vehicle and the extended states that comprise internal and external disturbances. The NLSEF compensates for the total disturbance to generate the control input. In the diagram, the state variable $\begin{array}{c} r_{1} \end{array}$ Here, the tracking signal of the target state $r_{1}={\left[\begin{array}{cc} v_{d} & \psi_{d} \end{array}\right]}^{\text{T}}$, Here, the state variable $r_{2}$ Here, the differential signal, $e_{1}$ and $e_{2}$ Here, the error signals, and $Z_{1}$, $Z_{2}$, and $Z_{3}$ are the values of the system states estimated by the ESO, where U is the control input, $y={\left[\begin{array}{cc} v & \theta \end{array}\right]}^{\text{T}}$ Here, the system output, and $b_{0}$ is the control parameter.

The TD uses the fastest feedback composite function to track changes in the input signal. This function typically has a high gain, causing it to be sensitive to measurement noise in the input signal. In actual operations of autonomous vehicles, sensors are affected by noise during measurements. Noise can severely affect the performance of a TD, leading to inaccurate or unstable output tracking and differentiation. A second-order sliding mode differentiator (2OSMD) is used to mitigate the impact of sensor noise and address the inaccuracies caused by noise in sensor measurement data.

**Fig 5:**
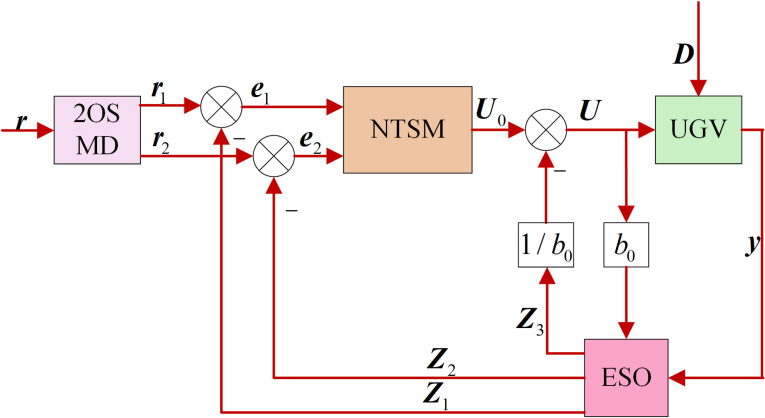
Structural block diagram of the 2OSMD-ADRC.

The 2OSMD-ADRC controller designed in this study replaces the TD with the 2OSMD to achieve high-quality state differentiation. The structure of the improved 2OSMD-ADRC is shown in Fig 5.

To smoothly track the target heading angle and speed under noisy conditions, the control system employs the 2OSMD. The expression for this differentiator is


$$\left\{ \begin{array}{c} @l{\dot{r}}_{1}=z_{1}\\ z_{1}=-\lambda_{1}|r_{1}-r(t){|}^{2/3}sgn(r_{1}-r(t))+z_{2}\\ {\dot{r}}_{2}=z_{2}\\ z_{2}=-\lambda_{2}|r_{2}-z_{1}{|}^{1/2}sgn(r_{2}-z_{1})+r_{3}\\ {\dot{r}}_{3}=-\lambda_{3}sgn(r_{3}-z_{2}) \end{array}\right.,$$ (40)

where $\begin{array}{c} r(t)=r_{o}(t)+n(t) \end{array}$; $\begin{array}{c} r_{o}(t) \end{array}$ represents the original signal; $\begin{array}{c} n(t) \end{array}$ represents the noise interference; $\begin{array}{c} r_{1} \end{array}$, $\begin{array}{c} r_{2} \end{array}$, and $\begin{array}{c} r_{3} \end{array}$ represent the zeroth-, first-, and second-order derivative estimates of $\begin{array}{c} r(t) \end{array}$, respectively; $z_{1}$ is the estimation error correction term for $\begin{array}{c} r(t) \end{array}$ and is used to update the zeroth-order estimate $\begin{array}{c} r_{1} \end{array}$; $z_{2}$ is the derivative estimation error correction term for $\begin{array}{c} r(t) \end{array}$ and is used to update the first-order estimate $\begin{array}{c} r_{2} \end{array}$; and $\lambda_{1}$, $\lambda_{2}$, and $\lambda_{3}$ are the design parameters of the differentiator. The 2OSMD can rapidly track the input signals with high precision and simultaneously obtain high-quality differential signals.

To estimate the various disturbances present during the control process, a linear ESO is established, and it can be expressed as


$$\left\{ \begin{array}{c} @le_{eso}=Z_{1}-y\\ {\dot{Z}}_{1}=Z_{2}-b_{01}e_{eso}\\ {\dot{Z}}_{2}=Z_{3}+b_{0}U-b_{02}fal(e_{eso},a_{1},\Delta_{1})\\ {\dot{Z}}_{3}=-b_{03}fal(e_{eso},a_{2},\Delta_{2}) \end{array}\right.,$$ (41)

where $\begin{array}{c} e_{eso} \end{array}$ represents the error between the observed and actual values of $\begin{array}{c} y \end{array}$; $Z_{1}$, $Z_{2}$, and $Z_{3}$ represent the observed values of y, $\dot{y}$, and the disturbances in the control system, respectively; and $b_{0}$, $b_{01}$, $b_{02}$, $b_{03}$, $a_{1}$, $a_{2}$, $\Delta_{1}$, and $\Delta_{2}$ are tunable parameters. The ESO can estimate the disturbances in the heading angle and velocity control systems in real time and effectively compensate for them, thereby improving the robustness and stability of the system under unknown disturbances.

The function fal(⬝) is defined as


$$fal\left(\rho ,\iota ,\sigma \right)=\left\{ \begin{array}{cc} {\left\|\rho \right\|}^{\iota }\text{sgn}(\rho ), & \left\|\rho \right\|>\sigma \\ \frac{\rho }{\sigma^{1-\iota }}, & ∥\rho ∥\leq \sigma \end{array}\right.,$$ (42)

where σ is the length of the linear interval and 0<ι<1. In nonlinear state error feedback, the system state errors are denoted as $e_{1}$ and $e_{2}$. Here, the controller's output U Here, the calculated based on the errors, and the nonlinear error feedback is expressed as


$$\left\{ \begin{array}{c} @le_{1}=r_{1}-Z_{1}\\ e_{2}=r_{2}-Z_{2}\\ U_{0}=k_{p}fal(e_{1},a_{3},h_{1})+k_{d}fal(e_{2},a_{4},h_{1})\\ U=U_{0}-\frac{Z_{3}}{b_{0}} \end{array}\right.,$$ (43)

where $k_{p}$, $k_{d}$, $a_{3}$, $a_{4}$, and $h_{1}$ are tunable parameters. Equation (43) ensures that under various working conditions, especially those that include other disturbances, the system error can converge rapidly and enable the desired control accuracy to be reached.

**Theorem 3**: For a given σ>0, if the values of $a_{1},a_{2},b_{0i},i=1,2,3$ are appropriately selected, the following conditions are satisfied:


$$\left\{ \begin{array}{c} @l0<a_{2}\leq a_{1}\leq 1\\ 0<b_{01}<b_{02}<b_{03}\\ b_{03}<b_{01}b_{02}\sigma^{\left(a_{1}-a_{2}\right)} \end{array}\right..$$ (44)

Subsequently, the estimation error of the ESO exponentially converges to a small neighborhood around the origin.

**Proof**:

Defining $\begin{array}{c} e_{eso\text{2}}=Z_{2}-\dot{y} \end{array}$, $\begin{array}{c} e_{eso3}=Z_{3}-D \end{array}$, and $\begin{array}{c} e_{0}={\left[e_{eso}\text{,}e_{eso2}\text{,}e_{eso3}\right]}^{\text{T}} \end{array}$, the estimation error $e_{0}$ is given as


$${\dot{𝐞}}_{0}=A_{0}\left(e_{eso}\right)e_{0}-\Gamma e_{eso\text{2}}+W,$$ (45)

$$\mathrm{\backslash [}\begin{array}{cc} & \#x0026;\det\left(sI-A_{0}\right)=s^{3}+b_{01}s^{2}+b_{02}|e_{eso}{|}^{-(1-a_{1})}s\\ & \#x0026;\;+b_{03}|e_{eso}{|}^{-(1-a_{2})}. \end{array}\mathrm{\backslash ]}$$ (46)

Based on the Routh criterion, if $b_{0i}>0,i=1,2,3$ and


$$b_{01}b_{02}|e_{eso}{|}^{-(1-a_{1})}>b_{03}|e_{eso}{|}^{-(1-a_{2})},$$ (47)

then Equation (46) is Hurwitz. The positive definite matrices $P_{0}$ and $Q_{\text{0}}$ satisfy


$$A_{0}^{\text{T}}P_{0}+P_{0}A_{0}\leq -Q_{0},$$ (48)

Let $E={\left[010\right]}^{\text{T}}$. Then


$$\mathrm{\backslash [}\begin{array}{cc} & \#x0026;{\left(A_{0}-\Gamma E\right)}^{\text{T}}P_{0}+P_{0}\left(A_{0}-\Gamma E\right)\\ & \#x0026;={A_{0}}^{\text{T}}P_{0}+P_{0}A_{0}-\gamma_{2,2}\leq -{Q_{0}}^{T}, \end{array}\mathrm{\backslash ]}$$ (49)

where $\gamma_{2,2}={\left(\Gamma E\right)}^{\text{T}}P_{0}+P_{0}\Gamma E$ and ${Q_{0}}^{\text{T}}=Q_{0}+\gamma_{2,2}$. By appropriately selecting $b_{0i},i=1,2,3$, ${Q_{0}}^{\text{T}}>0$ can be ensured. The Lyapunov function $V_{2}$ is expressed as


$$V_{2}=e_{0}^{\text{T}}P_{0}e_{0},$$ (50)

$$\begin{array}{c} {\dot{V}}_{2}=e_{0}^{\text{T}}\left({\left(A_{0}-\Gamma 𝐄\right)}^{\text{T}}P_{0}+P_{0}\left(A_{0}-\Gamma 𝐄\right)\right)e_{0}\\ +2e_{0}^{\text{T}}P_{0}\;\;\Psi \;\;\leq \gamma V_{2}+\varepsilon {\bar{\eta }}^{2}, \end{array}$$ (51)

where ε>0, $\gamma =\frac{\left(\lambda_{\min}\left({Q_{0}}^{\text{T}}\right)-\varepsilon^{-1}∥P_{0}{∥}^{2}\right)}{\lambda_{\max}\left(P_{0}\right)}$, and $\lambda_{\max}(*)$ and $\lambda_{\text{min}}(*)$ denote the maximum and minimum eigenvalues, respectively. If $b_{01}$, $b_{02}$, and $b_{03}$ satisfy $\lambda_{\text{min}}({Q_{0}}^{\text{T}})-\varepsilon^{-1}{\left\|P_{0}\right\|}^{2}>0$, then γ>0. Moreover,


$$\mathrm{\backslash [}V_{2}\left(t\right)\leq V_{2}\left(0\right)e^{-\gamma t}+\frac{1}{\gamma }\varepsilon {\bar{\eta }}^{2}.\mathrm{\backslash ]}$$ (52)

By appropriately selecting $b_{0i},i=1,2,3$, γ, and ε, the estimation error $e_{0}$ exponentially converges to a small neighborhood around the origin. If the three inequalities in Equation (44) are satisfied, Equation (52) holds. If $\begin{array}{c} \left|e_{eso}\right|\leq \delta \end{array}$, Equation (45) can be rewritten as


$${\dot{e}}_{0}=A_{0}\left(e_{eso}\right)e_{o}-\Gamma e_{eso2}+W.$$ (53)

Similarly, for the Lyapunov function $V_{3}=e_{0}^{\text{T}}P_{0}e_{0}$, the parameter $a_{1},a_{2},b_{0i},i=1,2,3$ can be shown to satisfy the inequality $b_{03}<b_{01}b_{02}\delta^{\left(a_{1}-a_{2}\right)}$, and the conclusion of Equation (51) can also be proven.

**Theorem 4**: If the error of the ESO exponentially converges to a small neighborhood around the origin, and the feedback gains of the control laws are $k_{p}>0$ and $k_{d}>0$, then the errors of the 2OSMD-ADRC algorithm in Equations (40)-(43) will asymptotically converge to zero, enabling $\psi_{d}$ and $v_{d}$ to be tracked in a short time.

**Proof**:

The dynamic model of the autonomous vehicle in Equation (10) can be simplified to:


$$\dot{\xi }=Bu+D.$$ (54)

The target tracking error is


$$\xi_{e}=\xi -\xi_{d}=\left[\begin{array}{c} @l@v-v_{d}\\ \dot{\theta }-{\dot{\psi }}_{d} \end{array}\right]=\left[\begin{array}{c} @l@v_{e}\\ {\dot{\psi }}_{e} \end{array}\right].$$ (55)

According to Equations (54) and (55), the system model of the autonomous vehicle can be expressed as


$${\dot{\xi }}_{e}=Bu+D-{\dot{\xi }}_{d},$$ (56)

By differentiating Equation (56), the system error dynamics can be derived as


$${\dot{\xi }}_{e}=A_{e}\xi +A_{X}\mu .$$ (57)

Choosing positive values for $\lambda_{i}<\lambda_{i+1}(i=1,2,3,4)$, we have


$$\left|\lambda I-A_{e}\right|=\prod_{i=1}^{5}\left(\lambda +\lambda_{i}\right).$$ (58)

The eigenvalues of $A_{e}$ are distinct; thus, an invertible real matrix K exists such that,


$${\text{e}}^{A_{e}t}=K\text{diag}\{e^{-\lambda_{1}t},{\text{e}}^{-\lambda_{2}t},{\text{e}}^{-\lambda_{3}t},{\text{e}}^{-\lambda_{4}t},{\text{e}}^{-\lambda_{5}t}\}K^{-1}.$$ (59)

The solution to the system error is


$$E(t)={\text{e}}^{A_{e}t}E(0)+\int_{0}^{t}{\text{e}}^{A_{e}(t-\tau )}A_{x}\mu (\tau )\text{d}\tau .$$ (60)

When t>0, applying the $m_{\infty }$ -norm to Equation (60) results in


$$∥{\text{e}}^{A_{e}t}{∥}_{m\infty }\leq \beta^{*}{\text{e}}^{-\lambda_{1}t}(t>0),$$ (61)

where $\beta^{*}$ is a constant. Furthermore, let $|Z_{3}|\leq \alpha^{*}$, where $\alpha^{*}$ is a constant; then,


$$\mathrm{\backslash [}\begin{array}{cc} & \#x0026;\left\|A_{X}\mu \right\|=\sqrt{{\left(-D+\frac{B}{b_{0}}Z_{3}\right)}^{2}+{\left(\frac{b_{0}-B}{b_{0}}Z_{3}\right)}^{2}+{\dot{D}}^{2}}.\\ & \#x0026;\leq N_{1}+N_{2}+\frac{B+|b_{0}-B|}{b_{0}}\alpha^{*} \end{array}\mathrm{\backslash ]}$$ (62)

Using the compatibility of the $m_{\infty }$ -norm with the 2-norm of the vectors in the complex domain,


$$\mathrm{\backslash [}\begin{array}{cc} & \#x0026;\left\|E(t)\right\|\leq {\text{e}}^{A_{e}t}E(0)+\int_{0}^{t}{\text{e}}^{A_{e}(t-\tau )}A_{X}\mu (\tau )\text{d}\tau \\ & \#x0026;\leq \beta^{*}\left\|E(0)\right\|+\frac{\beta^{*}}{\lambda_{1}}(N_{1}+N_{2}+\frac{B+|b_{0}-B|}{b_{0}}\alpha^{*})\\ & \#x0026;=N_{3}. \end{array}\mathrm{\backslash ]}$$ (63)

The proof is thus completed.

## 6. Simulation and experimental validation

### 6.1. Numerical simulation experiment

We validated the effectiveness of the proposed control method under various working conditions using a MATLAB R2022b/Simulink environment to construct a simulation model of the autonomous vehicle based on the dynamic and kinematic mathematical models established in Section 1. The motion behavior of the autonomous vehicle under complex dynamic conditions was accurately simulated by implementing these mathematical models in Simulink, providing reliable simulation support for controller debugging and optimization. The parameters used in the simulation are shown in Table 2.

**Table 2:** Parameters used in this simulation of the autonomous vehicle.

Parameter	Value	Parameter	Value
$k_{x},k_{y}$	1	$b_{01}$	25
$a_{x},a_{y}$	1	$b_{02}$	30
$k_{\vartheta_{x}},k_{\vartheta_{y}}$	0.5	$b_{03}$	100
$\epsilon_{\vartheta_{x}},\epsilon_{\vartheta_{x}}$	0.5	$b_{0}$	0.35
$\lambda_{1},\lambda_{2},\lambda_{3}$	25, 60, 500	$k_{p},k_{d}$	1.35, 5
$\beta_{1},\beta_{2},\beta_{3}$	30, 300, 1000	$h_{1}$, $\Delta_{1}$, $\Delta_{2}$	0.005
$a_{1},a_{2}$	0.5, 0.25	$a_{3},a_{4}$	0.5, 1.2

The parameters $k_{x}$ and $k_{y}$ in Table 2 are proportional gains for position error, used to adjust the response of the system to tracking errors. Larger values of $k_{x}$ and $k_{y}$ result in a faster response to errors; however, if the values are too large, they may cause system oscillations, affecting stability. Considering both tracking accuracy and system stability, we set $k_{x}=k_{y}=1$. The parameters $a_{x}$ and $a_{y}$ adjust response speed—controlling how quickly errors are eliminated. Larger values allow the system to eliminate errors more quickly but may cause overshoot and oscillations. To balance response speed and stability, we set $a_{x}=a_{y}=1$. The parameters $k_{\vartheta_{x}}$ and $k_{\vartheta_{y}}$ are stability gains for auxiliary variables, influencing the rate of change of internal system variables. Larger gains accelerate system stabilization but may introduce high-frequency oscillations, whereas smaller gains ensure a smoother response and enhance stability. We set $k_{\vartheta_{x}}=k_{\vartheta_{y}}=0.5$. The parameters $\epsilon_{\vartheta_{x}}$ and $\epsilon_{\vartheta_{y}}$ are error threshold parameters. Larger thresholds allow the system to maintain nonlinear control under significant errors, while smaller thresholds enable a transition to linear control for minor errors, enhancing stability. In this study, we set $\epsilon_{\vartheta_{x}}=\epsilon_{\vartheta_{y}}=0.5$. The parameters $\lambda_{1},\lambda_{2},\lambda_{3}$ determine tracking accuracy, error convergence speed, and system stability, respectively. A larger $\lambda_{1}$ enhances tracking accuracy but may cause oscillations, whereas a smaller value slows the response. We set $\lambda_{1}=25$ to balance these factors. $\lambda_{2}$ affects error convergence speed and system stability; larger values improve tracking precision but may induce oscillations, while smaller values slow the response. We set $\lambda_{2}=60$. $\lambda_{3}$ adjusts the smoothness of higher-order derivatives; a larger value suppresses high-frequency oscillations but affects tracking accuracy. We set $\lambda_{3}=500$. $\beta_{1},\beta_{2},\beta_{3}$ are observation parameters in Equation (17). Typically, $\beta_{1}>\beta_{2}>\beta_{3}$. We set $\beta_{1}=30$, $\beta_{2}=300$, and $\beta_{3}=1000$. According to Theorem 3, the parameter values for $b_{01}$, $b_{02}$, $b_{03}$ are set as follows: $b_{01}=25$, $b_{02}=30$ and $b_{03}=100$. $b_{0}$ is a proportional gain used to adjust the output range of the control signal. We set $b_{0}=0.35$. $k_{p}$ is a proportional feedback parameter that adjusts control output based on current error size. In this experiment, we set $k_{p}=1.35$. $k_{d}$ is an integral feedback parameter that introduces an integral term to control errors based on their magnitude. In this experiment, we set $k_{d}=5$. Larger values of parameters $a_{1}$ and $a_{3}$ can enhance error convergence speed but may cause system oscillations, while smaller values slow down the response. In this study, we set $a_{1}=a_{3}=0.5$. Parameters $a_{2}$ and $a_{4}$ affect the observation accuracy of higher-order states; larger values improve convergence speed but may induce high-frequency oscillations, while smaller values result in a slower response. We set $a_{2}=0.25$, $a_{4}=1.2$. Parameters $h_{1}$, $\Delta_{1}$, and $\Delta_{2}$ are smoothing factors for the nonlinear function; excessively large values weaken the nonlinear effect, while excessively small values increase the risk of high-frequency jitter. We set $h_{1}=\Delta_{1}=\Delta_{2}=0.005$.

#### 6.1.1. Experiment 1: Effectiveness of the odometry compensation strategy.

**Fig 6:**
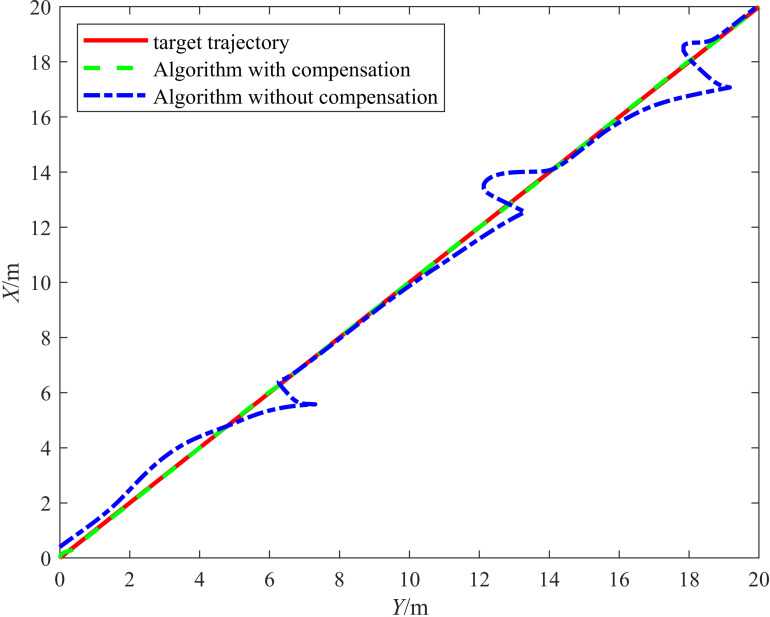
Comparison between the target tracking before and after the odometry compensation was applied.

In the simulation, the disturbances affecting the odometry in the *X* and *Y* directions were set as $f_{x}=\sin(\frac{\;\;\pi \;\;}{5}t)$ and $f_{y}=-2+\frac{2}{3}\text{(t}-\text{6k) },6k\leq t<6(k+1)$, respectively (where k=0,1,2⋯), and the target trajectory was set as $\left\{ \begin{array}{c} x_{r}=t\\ y_{r}=t \end{array}\right.$. A comparative experiment was conducted before and after introducing the odometry compensation strategy. The experimental results are shown in Fig 6, in which the solid line represents the target’s trajectory, the dash-dotted line represents the vehicle’s trajectory before the odometry compensation strategy was applied, and the dashed line represents the vehicle’s trajectory after the odometry compensation strategy was applied. As shown in Fig 6, applying the odometry compensation strategy significantly improved the target tracking.

**Fig 7:**
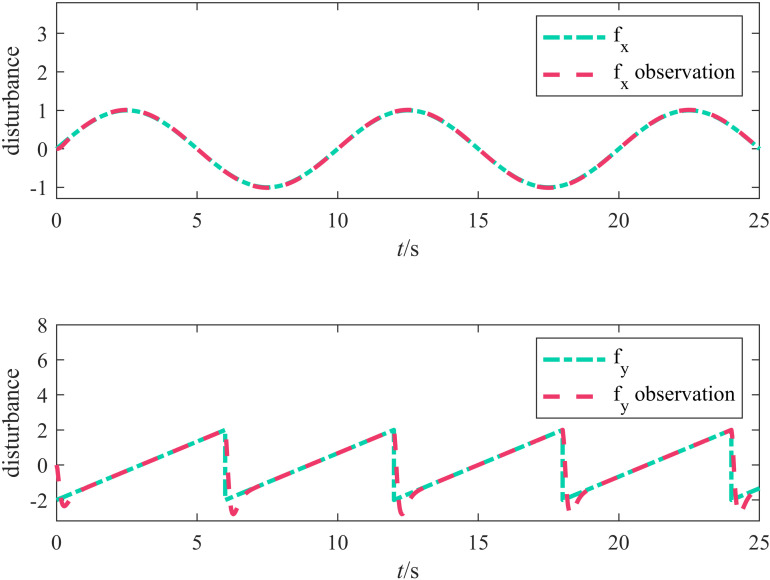
ESO disturbance observation curve.

Fig 7 illustrates the ESO disturbance observation curve; the dash-dotted line represents the actual disturbance experienced by the odometer, and the dashed line represents the disturbance observed by the ESO. The figure indicates that the disturbance in the *X* direction changed smoothly and that the ESO’s observed values stably followed the actual disturbance curve, indicating that the ESO effectively estimated the disturbances affecting the odometry. In the *Y* direction, the disturbance changes were more severe, and slight discrepancies between the observed and actual values of the ESO were observed; however, the overall error remained small.

**Fig 8:**
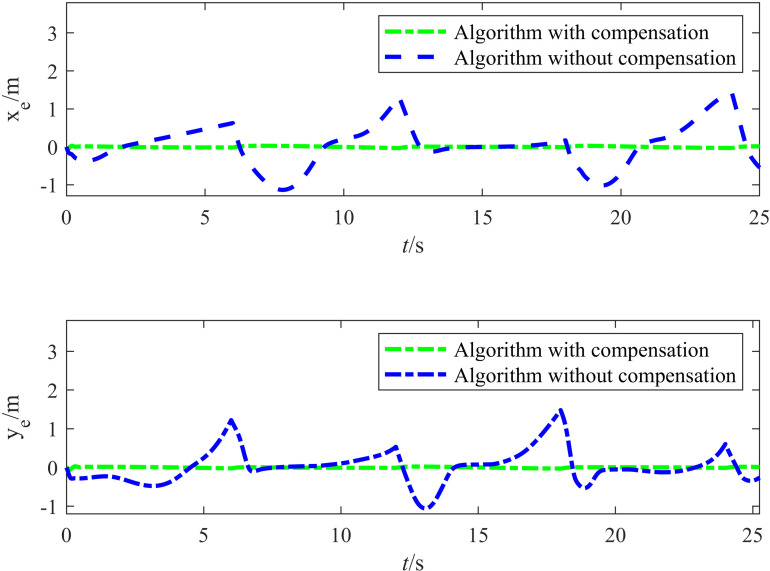
Tracking error diagram for before and after applying the odometry compensation.

Fig 8 presents the target tracking error of the vehicle. The target tracking error in the *X* direction was small and could be ignored. Although the odometry experienced severe disturbance variations in the *Y* direction, the target tracking error in the *Y* direction indicates that the target tracking of the vehicle only began to deviate during a disturbance spike at approximately 6 s. Under the influence of the controller, the trend in the target tracking error was suppressed within 3 s, and the error converged. The maximum error in the *Y* direction (0.03 m) demonstrates that the controller effectively compensated for disturbances.

Table 3 lists the error evaluation metrics with and without odometry compensation, including the root mean square error (RMSE) and maximum error. The position errors $e_{x}$ and $e_{y}$ were measured in meters (m), and the angular error $e_{\theta }$ was measured in radians (rad). Table 3 indicates that without the compensation algorithm, both the RMSE and maximum error exhibited large values. In particular, the maximum errors in the *X* and *Y* directions were 1.4207 m and 1.4814 m, respectively, and the maximum error in the heading angle was 1.0985 rad. This indicates that under complex working conditions, the accumulation of odometry errors had a significant impact on the target tracking of the vehicle, resulting in severe deviations in both position and heading. In contrast, after introducing the compensation algorithm, the RMSE and maximum error of the system were significantly reduced. Specifically, the RMSE in the *X* and *Y* directions decreased to 0.0152 m and 0.0124 m, respectively, and the corresponding maximum errors decreased to 0.0439 m and 0.0602 m, respectively. This demonstrated the effectiveness of the compensation strategy in suppressing the accumulation of odometry errors. Although the maximum heading angle error was 0.6170 rad, it was still reduced by approximately 43.8% compared to the uncompensated case, which further illustrates how effectively the ESO compensates for odometry errors in real time in complex dynamic environments. In summary, the experimental results validated how the compensation strategy significantly improved the target tracking accuracy and robustness, which enabled the autonomous vehicle to more precisely track the target in complex environments.

**Table 3:** Error evaluation index with and without odometry compensation.

Control Methods	$||e_{x}|{|}_{\text{rms}}$	$||e_{y}|{|}_{\text{rms}}$	$||e_{\theta }|{|}_{\text{rms}}$	$||e_{x}|{|}_{\max}$	$||e_{y}|{|}_{\max}$	$||e_{\theta }|{|}_{\max}$
Algorithm without compensation	0.5586	0.4270	0.5102	1.4207	1.4814	1.0985
Algorithm with compensation	0.0152	0.0124	0.0645	0.0439	0.0602	0.6170

#### 6.1.2 . Experiment 2: Validation of the rapidness of the HLOS guidance strategy.

**Fig 9:**
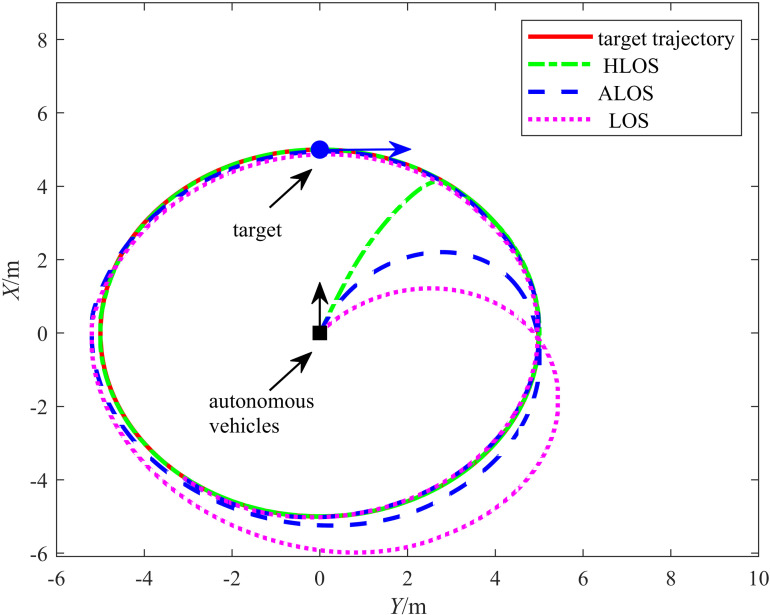
Target tracking graph for a circular trajectory.

Comparative experiments were conducted on the proposed HLOS strategy, traditional LOS strategy, and adaptive LOS (ALOS) strategy from [19] to validate the effectiveness of the proposed HLOS strategy. In all cases, the control algorithm was the ADRC algorithm. The target trajectory was set as a circle with a motion equation of $\left\{ \begin{array}{c} x_{r}(t)=5\cos(0.4t)\\ y_{r}(t)=5\sin(0.4t) \end{array}\right.$, and the linear and angular velocities were set as $v_{r}=2\text{ m/s}$ and $\omega_{r}=0.4\text{ rad/s}$, respectively. The initial positions of the target and autonomous vehicle were $\left(x_{r},y_{r},\theta_{r})=(5,0,\frac{\;\;\pi \;\;}{2}\right)$ and $\left(x_{0},y_{0},\theta_{0})=(0,0,0\right)$, respectively, with an initial separation of 5 m. A comparison between the target tracking performances of the three methods is shown in Fig 9. The solid, dash-dotted, dashed, and dotted lines represent the trajectories of the target, proposed HLOS strategy, ALOS strategy from [19], and traditional LOS strategy, respectively. According to Fig 9, the proposed HLOS, ALOS, and traditional LOS strategies accomplished the target tracking task. The ALOS strategy exhibited significant deviations from the target at the trajectory’s turning points, leading to larger tracking errors. The traditional LOS strategy exhibited apparent deviations at sharp turns owing to the limitations of a fixed look-ahead distance. The proposed method tracked the target more smoothly and stably than the other two strategies. In the proposed HLOS strategy, the tanh(x) function effectively suppressed excessive corrections of large errors, allowing the autonomous vehicle to maintain stable tracking even during sharp turns.

**Fig 10:**
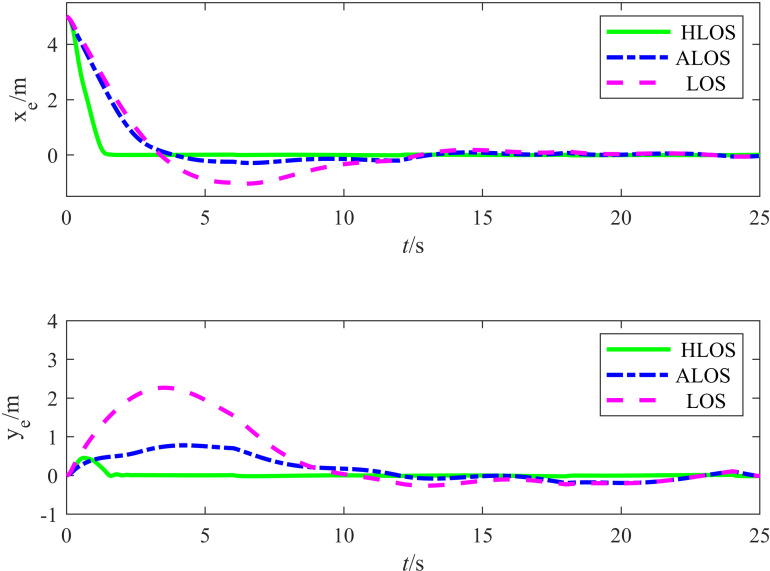
Plot comparing the circular tracking errors of the three algorithms.

Fig 10 shows the simulation results for the target tracking errors $x_{e}$ and $y_{e}$, illustrating the error convergence exhibited by the three algorithms during tracking. According to Fig 10, the proposed algorithm rapidly converged the errors in both the *X* and *Y* directions. However, the ALOS and traditional LOS strategies converged at slower rates and with larger error fluctuations. Therefore, the error curves indicate that the error convergence of the proposed HLOS strategy exhibited superior speed and stability.

**Fig 11:**
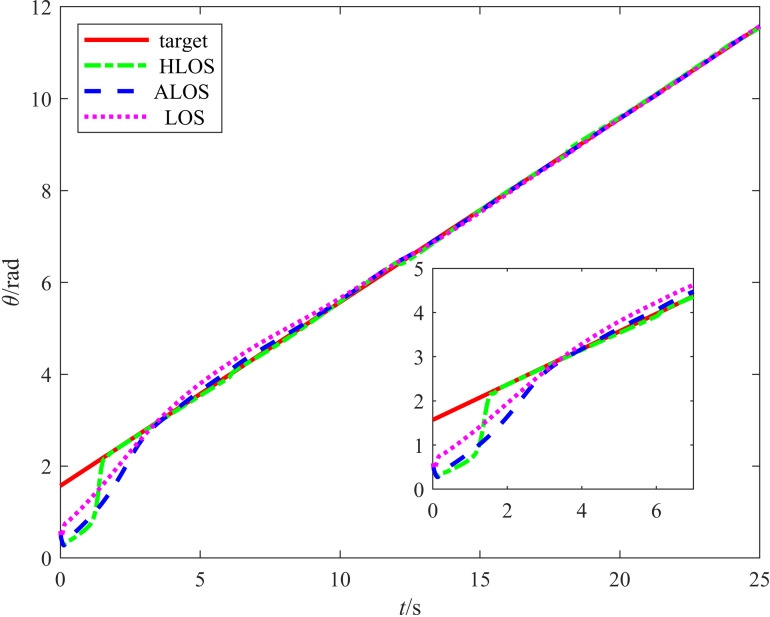
Plot comparing the circular angle tracking performances of the three algorithms.

The angle tracking results are shown in Fig 11, which displays the target heading angle tracking curves for the proposed HLOS, ALOS, and traditional LOS strategies. For a clearer view of the response speed and accuracy, the inset shows the heading angle variations in the early stages. According to Fig 11, the proposed strategy significantly outperformed the ALOS and traditional LOS strategies in angle tracking. In the initial stage, the HLOS strategy responded quickly and achieved a high alignment rate with the target heading angle, the ALOS strategy exhibited slower angle responses with larger deviations, and the traditional LOS strategy exhibited even slower responses with more pronounced lag. The HLOS strategy exhibited higher sensitivity in dynamic responses, enabling it to quickly adapt to changes in the target angle, whereas the ALOS and traditional LOS strategies exhibited sluggish responses with significant lag. Thus, the proposed HLOS strategy demonstrated significant advantages in dynamic adjustment and angle control.

Table 4 lists the error evaluation metrics for the three strategies, including the RMSE and maximum error. The position errors $e_{x}$ and $e_{y}$ were measured in m, while the angular error $e_{\theta }$ was measured in rad. According to Table 4, the proposed method outperformed the ALOS and traditional LOS strategies in terms of the RMSE and maximum error. For the RMSE of the position in the *X* and *Y* directions, the error reduction rate for the ALOS strategy was 41.50%, while the proposed method achieved a reduction rate of 96.52%. For the θ angular error, the ALOS strategy achieved a reduction rate of 28.40%, while the proposed method reached a reduction rate of 40.61%. Regarding the maximum error, the proposed method exhibited lesser maximum errors in the *X* and *Y* directions and reduced angular measurements compared to the ALOS and traditional LOS methods, demonstrating a significant improvement in accuracy.

**Table 4:** Results for the error evaluation indices of the three strategies.

Control Methods	$||e_{x}|{|}_{\text{rms}}$	$||e_{y}|{|}_{\text{rms}}$	$||e_{\theta }|{|}_{\text{rms}}$	$||e_{x}|{|}_{\max}$	$||e_{y}|{|}_{\max}$	$||e_{\theta }|{|}_{\max}$
LOS	0.1322	0.2994	0.0426	0.2906	0.7787	0.1119
ALOS	0.0920	0.1605	0.0305	0.1824	0.2621	0.0646
HLOS	0.0061	0.0089	0.0253	0.0132	0.0321	0.0495

#### 6.1.3. Experiment 3: Validation of the controller’s noise resistance capability.

**Fig 12:**
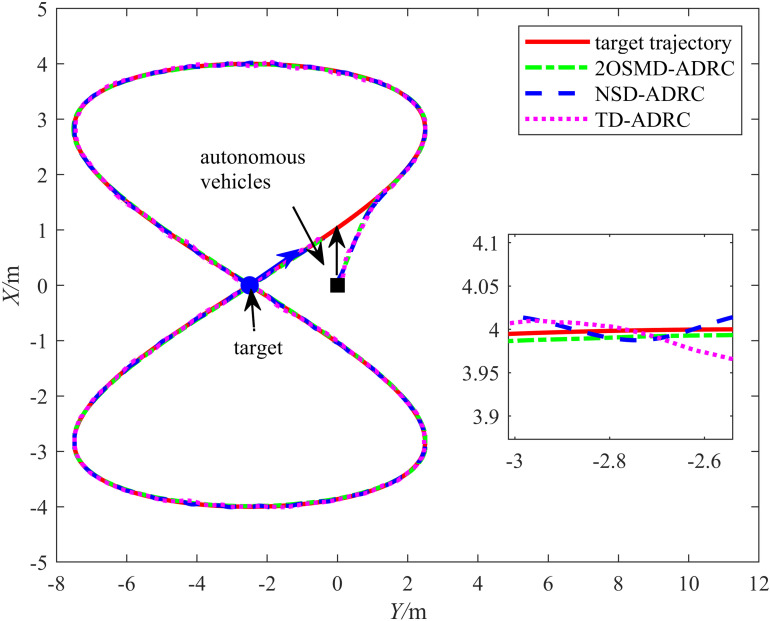
Chart showing the target tracking performances of the three control algorithms under noise.

A comparative experiment was conducted using the TD and the noise sampling differentiator (NSD) proposed in [25] to validate the noise resistance performance of the proposed 2OSMD-ADRC. The proposed HLOS strategy was used as the guidance strategy for the target tracking method, and the control algorithms used were 2OSMD-ADRC, TD-ADRC, and NSD-ADRC. The target trajectory was $\begin{array}{c} \left\{ \begin{array}{cc} & \;\;\;\;\;\;\;\;x_{r}(t)=4\sin(\text{0.1625}t)\\ & \;\;\;\;\;\;\;\;y_{r}(t)=5\sin(\text{0.325}t)-2.5 \end{array}\right. \end{array}$, and the initial positions of the target and autonomous vehicle were $\left(x_{r},y_{r},\theta_{r})=(0,-2.5,0.38\;\;\pi \;\;\right)$ and $\left(x_{0},y_{0},\theta_{0})=(0,0,0\right)$, respectively. The initial distance between them was 2.5 m. The input signal was subjected to white noise n(t) during the experiment with a mean of 0 and a variance of 0.01. The results of the comparative target tracking experiment conducted under noise interference for the three controllers are illustrated in Fig 12. A comparison between the target tracking results of the different methods revealed that the 2OSMD-ADRC method accurately tracked the target trajectory and almost perfectly followed the target path under noise interference. In sections with significant trajectory changes, 2OSMD-ADRC responded quickly and effectively to suppress the noise interference. However, TD-ADRC and NSD-ADRC exhibited larger deviations between the actual and target trajectories when faced with complex trajectory changes. Moreover, under noise interference, the deviations became more pronounced, particularly during abrupt changes in the target trajectory, at which points the system failed to adjust in time, resulting in a significant lag. Overall, 2OSMD-ADRC demonstrated vastly superior trajectory tracking capabilities in noisy environments compared to the other methods.

**Fig 13:**
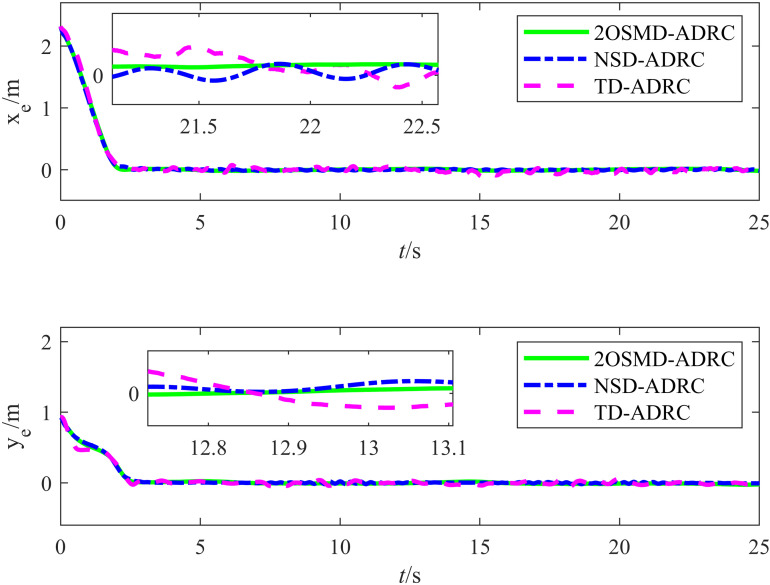
Graph showing the tracking errors of the three control algorithms under noise.

Fig 13 shows the tracking errors of the three controllers under noise interference. In the experiment, the input signal was subjected to white noise with a mean of 0 and variance of 0.01, and the tracking errors of the three differentiators exhibited various performance results in the *X* and *Y* directions. As shown in the figure, the proposed 2OSMD-ADRC method exhibited the smallest error fluctuation under noise interference, particularly in the local details of the errors. It demonstrated significant stability and was almost unaffected by the noise. In contrast, the TD-ADRC had larger error fluctuations in the *X* and *Y* directions, especially in the initial stages, where the error deviation was significant. Some fluctuations persisted even in the later stages, which is when it tended to stabilize. This indicates that the TD-ADRC had relatively poor stability in handling noise disturbances, as evidenced by its response lag. The NSD-ADRC method performed slightly better than TD-ADRC under noise interference. However, the error fluctuations were still significant overall, particularly when the noise was stronger, leading to larger error amplitudes and relatively low system robustness. Therefore, the 2OSMD-ADRC method demonstrated stronger noise resistance and higher system stability in noisy environments.

Table 5 lists the error evaluation metrics used to assess the control accuracy and robustness of the three controllers in noisy environments. According to Table 5, the error performances of the three controllers in terms of position and angle tracking were significantly different. TD-ADRC and NSD-ADRC exhibited lower tracking accuracy when faced with white noise interference, while the proposed method demonstrated significant advantages in handling white noise interference. The RMSE of the position errors in the *X* and *Y* directions for TD-ADRC were 0.0292 m and 0.0158 m, respectively. The RMSE of the *X* and *Y* position errors for NSD-ADRC were 0.0075 m and 0.0051 m, respectively, and the RMSE of the *X* and *Y* position errors for the proposed method reached 0.0023 m and 0.0025 m, respectively. Regarding the tracking accuracy in the *X* direction, the proposed method decreased the error by approximately 89.78% compared to that of TD-ADRC and by 63.49% compared to that of NSD-ADRC. These results indicate that the proposed method demonstrated significantly better robustness in handling the position errors than the other methods. Regarding the angular error RMSE, TD-ADRC and NSD-ADRC achieved 0.1613 rad and 0.1576 rad, respectively, while the proposed method achieved 0.1451 rad. Although the improvement in the angular error control was less pronounced than that for the position errors, it still enhanced the accuracy. For the maximum error in the *X* direction, the proposed method achieved 0.0068 m, which is a significant improvement compared to the 0.1181 m achieved by TD-ADRC and the 0.0192 m achieved by NSD-ADRC. The proposed method also exhibited smaller maximum errors in the *Y* direction and smaller angular measurements. According to the analysis above, the proposed method exhibited superior tracking performance in handling white noise interference, demonstrating excellent robustness and accuracy.

**Table 5:** Error evaluation indices for the three kinds of differentiators.

Control Methods	$||e_{x}|{|}_{\text{rms}}$	$||e_{y}|{|}_{\text{rms}}$	$||e_{\theta }|{|}_{\text{rms}}$	$||e_{x}|{|}_{\max}$	$||e_{y}|{|}_{\max}$	$||e_{\theta }|{|}_{\max}$
TD-ADRC	0.0292	0.0158	0.1613	0.1181	0.0596	1.1906
NSD-ADRC	0.0075	0.0051	0.1576	0.0192	0.0190	1.1904
2OSMD-ADRC	0.0023	0.0023	0.1451	0.0068	0.0053	1.0495

#### 6.1.4. Experiment 4: Comparison of control methods under complex conditions.

**Fig 14:**
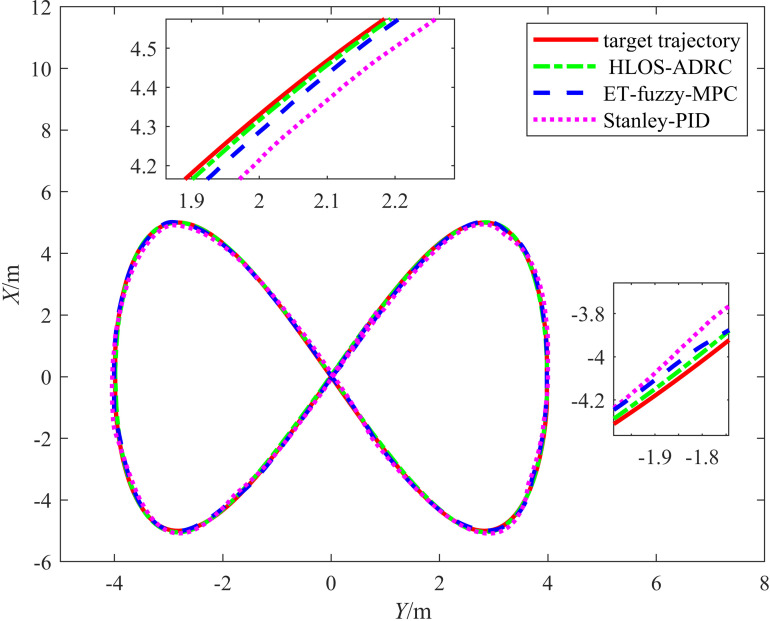
Diagram showing the double newline target tracking for the three control methods.

We further validated the robustness of the proposed control method through a comparative experiment on the Stanley-PID control method from [15] and the event-triggered fuzzy adaptive optimization MPC method (ET-Fuzzy-MPC) from [16]. During the experiment, the input signal was subjected to white noise $\begin{array}{c} n(t) \end{array}$ with a mean of 0 and a variance of 0.01, and the odometry was disturbed in the *X* and *Y* directions by $f_{x}=\sin(\frac{\;\;\pi \;\;}{5}t)$ and $f_{y}=-2+\frac{2}{3}\text{(}t-\text{6k) },6k\leq t<6(k+1)$, respectively (where k=0,1,2⋯). The target’s trajectory was a lemniscate curve, of which the equations of motion were $\begin{array}{c} \left\{ \begin{array}{cc} & \;\;\;\;\;\;\;\;x_{r}(t)=5\sin(\text{0.325}t)\\ & \;\;\;\;\;\;\;\;y_{r}(t)=4\sin(\text{0.1625}t)-2.5 \end{array}\right. \end{array}$. A comparison between the target tracking trajectories is shown in Fig 14, which shows the target trajectory, the trajectory of the proposed method, and the trajectories of the methods from [15,16]. When the odometry was affected by external disturbances, the vehicle could not obtain the actual deviation in the trajectory. Particularly when the vehicle was driving on curved roads, the speed variations of the left and right wheels were significant and inconsistent, causing the deviation error calculated using the odometry to deviate significantly from the actual deviation error. During target tracking, neither the Stanley-PID nor ET-Fuzzy-MPC algorithms compensated for the odometry data, resulting in certain deviations between the vehicle's trajectory and the target's trajectory for both algorithms. In contrast, the proposed ADRC control method compensated for the disturbances in the odometry, mitigating the effects of the external environment on the vehicle. Fig 14 indicates that the vehicle's actual driving trajectory is consistent with the target's trajectory.

**Fig 15:**
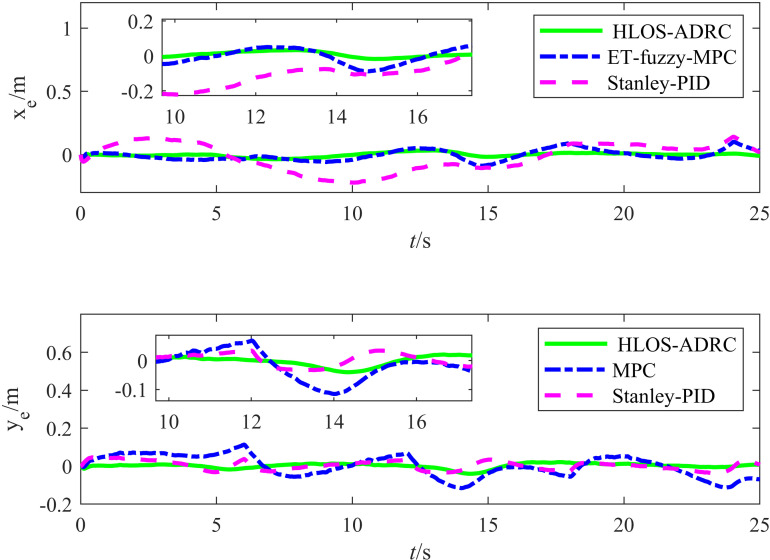
Diagram showing the double newline tracking errors for the three control methods.

Fig 15 illustrates the simulation results for the target tracking errors $x_{e}$ and $y_{e}$ for the lemniscate trajectory. According to Fig 15, the proposed method exhibited tracking errors in the *X* and *Y* directions that significantly differed from those of the Stanley-PID and ET-Fuzzy-MPC methods. The proposed method quickly converged in the *X* direction, demonstrating superior stability. In contrast, the Stanley-PID and ET-Fuzzy-MPC methods exhibited larger error fluctuations, significantly slower convergence speeds, and substantial oscillations in the initial stage. The proposed method also demonstrated faster error convergence in the *Y* direction, while the Stanley-PID and ET-Fuzzy-MPC methods exhibited larger initial errors. Although the error fluctuations eventually converged, they were significant throughout the process, indicating poor stability. Overall, the proposed method was more efficient at controlling the errors in both directions than the Stanley-PID and ET-Fuzzy-MPC methods, as demonstrated by its quicker error reduction speed and relatively high stability.

Table 6 presents the error evaluation metrics, including the RMSE and maximum error, for the Stanley-PID, ET-Fuzzy-MPC, and proposed methods. According to Table 6, the proposed method achieved a significantly lower RMSE and maximum error than the other methods. For the positional RMSE, the average values were 0.0859 m for Stanley-PID, 0.0492 m for ET-Fuzzy-MPC, and 0.0155 m for the proposed method. The data show that the proposed method achieved significant accuracy improvements in the positional RMSE, achieving a value lower than that of Stanley-PID and ET-Fuzzy-MPC by 82.01% and 68.61%, respectively, while maintaining smaller fluctuations. Regarding the angular error, the RMSE of the proposed method was lower than that of Stanley-PID and ET-Fuzzy-MPC, and it also exhibited better control accuracy. As shown in Table 6, the maximum errors in x, y, and θ for the proposed method were significantly smaller than those for the other methods. These results indicate that the proposed method exhibited high reliability and stability in complex environments.

**Table 6:** Error evaluation indices for the three methods.

Control Methods	$||e_{x}|{|}_{\text{rms}}$	$||e_{y}|{|}_{\text{rms}}$	$||e_{\theta }|{|}_{\text{rms}}$	$||e_{x}|{|}_{\max}$	$||e_{y}|{|}_{\max}$	$||e_{\theta }|{|}_{\max}$
Stanley-PID	0.1159	0.0558	0.0594	0.2565	0.1425	0.1782
ET-Fuzzy-MPC	0.0505	0.0479	0.0457	0.0877	0.1158	0.1473
The Proposed Method	0.0207	0.0102	0.0318	0.0474	0.0295	0.1205

Simulation tests were conducted on the experimental platform to further evaluate the computational efficiency of the proposed 2OSMD-ADRC method. The experimental computer was equipped with an i5-11300H quad-core processor and Windows 11, and the tests were conducted in the MATLAB R2022b/Simulink environment. Table 7 presents the average computation time per control step. The table also reveals that MPC requires solving multiple predictive optimization steps per control cycle, prolonging its computation time per step compared to that of 2OSMD-ADRC. Compared to ET-Fuzzy-MPC, the proposed method reduces computation time per step by 40.3%, verifying its higher computational efficiency. This improvement is due to the proposed method's reliance on real-time disturbance estimation via ESO rather than computationally intensive optimization, enabling faster execution and better real-time performance. Additionally, the proposed method reduces computation time per step by 38.4% compared to Stanley-PID. This improvement is primarily due to Stanley-PID’s lower function call frequency, which increases the processing load per step. Conversely, 2OSMD-ADRC operates at a higher frequency, distributing computations across more control steps, thereby reducing the computational load per step and achieving higher efficiency.

**Table 7:** Computation time per control step for the three methods.

Control Methods	Computation Time per Step/ms
Stanley-PID	0.007806
ET-Fuzzy-MPC	0.008048
The Proposed Method	0.004805

### 6.2. Real vehicle road test experiment

**Fig 16:**
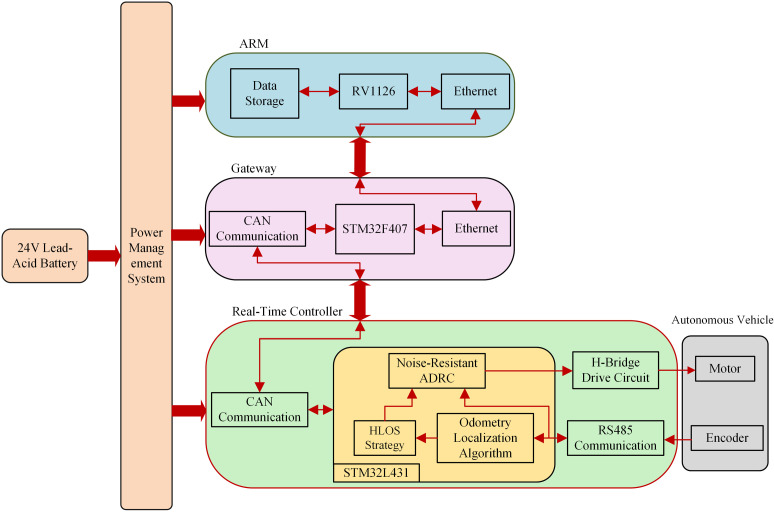
The unmanned vehicle experiment platform.

The system architecture of the autonomous vehicle experimental platform, which is based on the proposed algorithm, is illustrated in Fig 16. This motion control system adopts a hybrid hierarchical control architecture, integrating a central processor while retaining a distributed low-level communication framework. The overall system framework (Fig 16) consists of four components: the power management system, real-time controller, gateway, and ARM module.

The Advanced RISC Machine (ARM) module uses the RV1126 chip produced by Rockchip as the main control chip. This chip is based on a quad-core ARM Cortex-A7 architecture and integrates a 2T NPU. It primarily issues movement commands to the real-time controller, receives feedback from the controller, and stores the data in memory. In the system architecture, the STM32F407ZET6 microprocessor serves as the gateway processor, and it mainly handles communication protocol conversion between the ARM processor and the real-time controller. The microprocessor runs on the real-time operating system UCOS-II and supports the LwIP network protocol stack, ensuring stable and reliable communication. UCOS-II provides comprehensive support for task management, memory management, interrupt control, and clock management. However, the LwIP network protocol stack enables the handling of TCP/IP protocols, ensuring efficient data transmission and network communication. The real-time controller is the core component of the real-world vehicle experiments where the proposed method is executed. This module is responsible for receiving data from the encoder and compensating for it using the odometry-based localization algorithm to reduce cumulative errors. The extended state observer performs real-time disturbance estimation and correction, yielding optimized position data. This information is subsequently fed into the hyperbolic tangent Line-of-Sight guidance algorithm to compute the desired heading angle and speed commands. The Active Disturbance Rejection Controller further processes the computed results to generate motor control signals. The control signals are transmitted to the motor via the H-bridge driver circuit to regulate the vehicle's motion. Meanwhile, feedback signals from the actuators are transmitted back to the control system via RS485 communication for closed-loop adjustments, while the computed data is uploaded to the host computer via the CAN bus.

**Fig 17:**
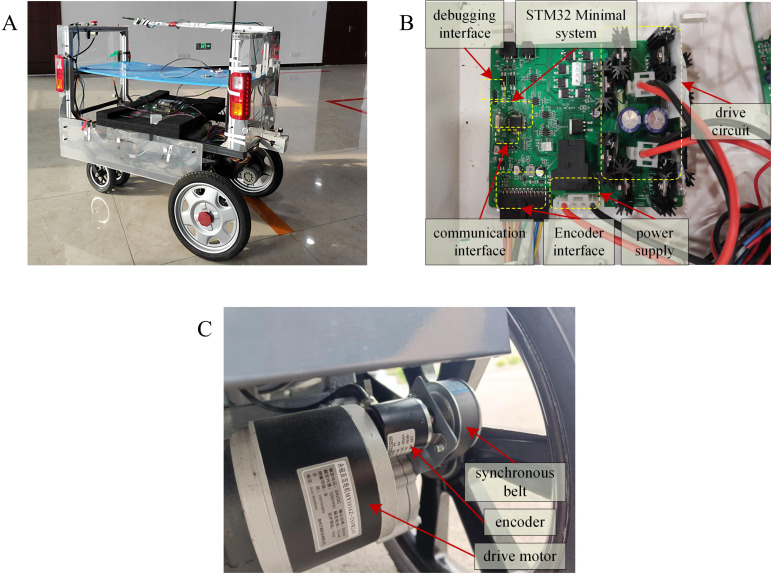
Experimental setup of the actual vehicle test. (A) Experimental vehicle. **(B)** Circuit board control. **(C)** Power detection devices.

The two-wheel drive differential autonomous vehicle used in the experiment is shown in Fig 17A. The vehicle features a lightweight aluminum alloy frame weighing 50 kg and having a 110 cm × 60 cm × 80 cm dimension. The core driving system is powered by two independently driven MY1016Z-250W 24V DC motors, each with a rated power of 250 W, rated current of 13.4 A, rated speed of 105 r/min, and a maximum torque of 20.45 N·m. The front wheels are the driving wheels, achieving differential drive through independently controlled motors. Fig 17B illustrates the control circuit built with a microcontroller (STM32L431RCT6) operating at a main frequency of 80 MHz. An H-bridge composed of metal-oxide-semiconductor field-effect transistors (MOSFETs) served as the motor driver circuit, and a 12-bit precision magnetoelectric encoder was used as the odometer. The encoder communicated with the controller using the Modbus protocol. The encoder was connected to the axle via a timing belt to ensure that it accurately recorded the actual distance traveled by the wheel. The installation positions of the motors and encoder are illustrated in Fig 17C.

We verified the anti-interference capability of the proposed control method by applying it to the experimental platform and conducting driving experiments under sideslip and uneven load interference conditions.

#### 6.2.1. Experiment 1: Vehicle anti-sideslip driving test.

**Fig 18:**
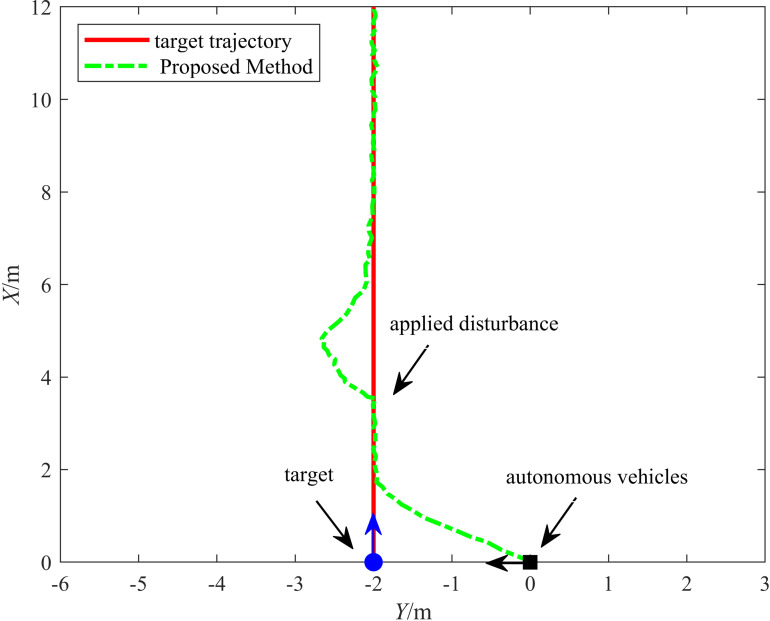
Anti-skid driving experiment.

This experiment validated the anti-sideslip capability of the proposed control method by simulating sideslip through an external lateral displacement, allowing us to assess tracking stability and path correction under external disturbances. Fig 18 illustrates the target tracking trajectory of the vehicle under disturbance conditions. The solid red line represents the target trajectory, while the dash-dotted green line represents the vehicle's tracking trajectory under the proposed control strategy. The figure reveals that the vehicle experiences lateral deviation when sideslip disturbance is applied. However, the control strategy swiftly counteracts the disturbance, allowing the vehicle to return to the planned path with minimal deviation.

**Fig 19:**
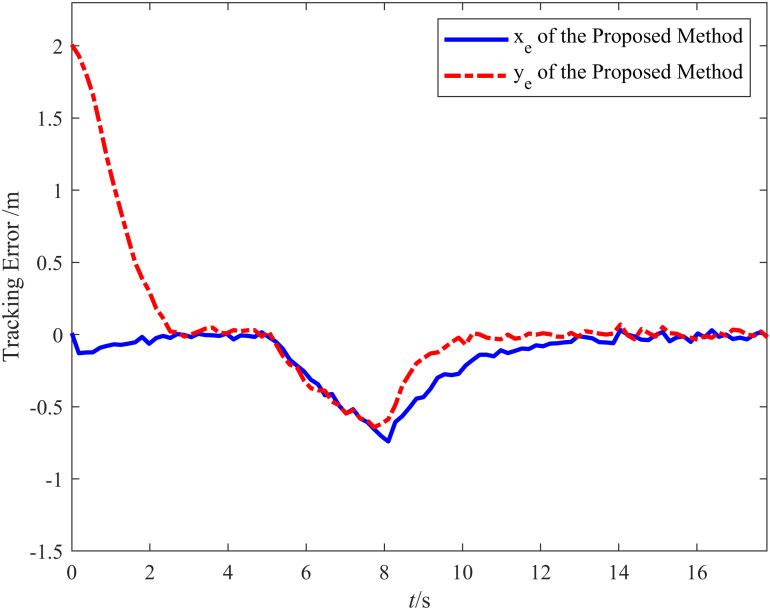
Trajectory deviation in anti-skid driving experiment.

Fig 19 illustrates the variation of lateral and longitudinal tracking errors over time in the anti-sideslip driving experiment. When the tracking begins, the Wheeled Mobile Robot immediately follows the target within 2.88 s under the proposed method, with minimal tracking error. When the disturbance is introduced, the lateral tracking error increases considerably. However, after approximately 4 s of adjustment, the error stabilizes, with the lateral and longitudinal tracking errors approaching zero and the error range converging within 0.069 m. This verifies that the vehicle has strong recovery capability after experiencing external disturbances, effectively reducing tracking errors and returning to the planned path.

#### 6.2.2. Experiment 2: Vehicle driving test under uneven load conditions.

**Fig 20:**
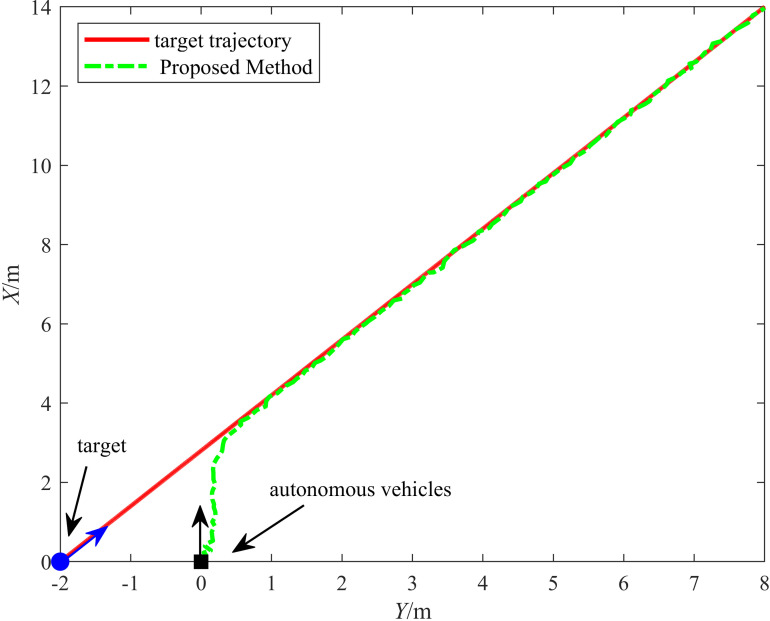
Driving experiment under uneven load conditions.

During vehicle operation, an unbalanced load inevitably shifts the center of gravity, resulting in unequal loads on the left and right wheels, deviating the vehicle from the target trajectory. We validated the effectiveness of the proposed control method under these conditions by loading an additional 30 kg into the right cargo box of the vehicle, causing a shift in the center of gravity. The vehicle's driving control performance is presented in Fig 20, where the solid line represents the target trajectory, while the dash-dotted line represents the actual driving trajectory. The proposed method effectively compensates for the deviation caused by the load imbalance and ensures stable target tracking. When the vehicle begins to move, it experiences significant lateral drift to the right due to the shift in the center of gravity. However, the control system adapts to the disturbance, and the proposed method successfully corrects the trajectory. This enables the vehicle to realign with the target, verifying the real-time disturbance rejection capability of the proposed method.

**Fig 21:**
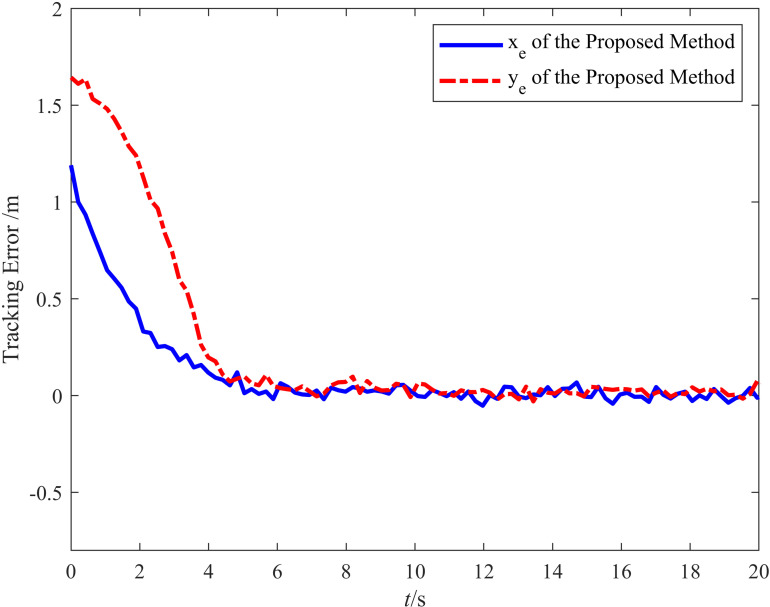
Trajectory deviation in uneven load driving experiment.

Fig 21 illustrates the variation of lateral and longitudinal errors over time in the uneven load-driving experiment. The figure reveals that the error converges to near zero at approximately 6 s under uneven load conditions, with a steady-state error stabilized at 0.14 m. This confirms that the proposed method effectively suppresses trajectory deviations caused by load imbalance.

We further validated the robustness of the proposed control method by applying it alongside the ET-Fuzzy-MPC control method from [16] to the same experimental platform. We evaluated it on the same road surface in vehicle experiments. The control performance for both straight-line and curved-path driving was assessed. The control performance of the vehicle was evaluated separately for straight-line, curve, and sharp-turn driving.

#### 6.2.3. Experiment 3: Vehicle straight-line driving test.

**Fig 22:**
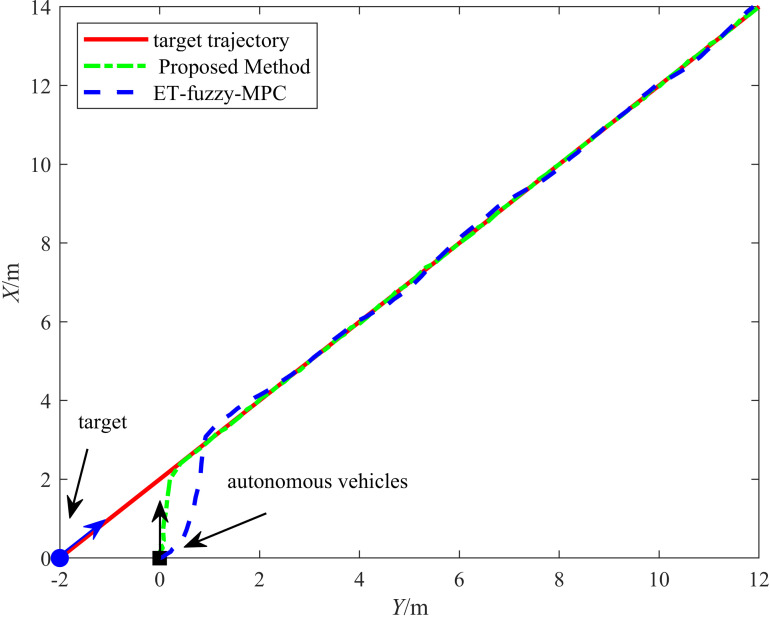
Results of the straight-line driving experiment.

The straight-line driving trajectories of the two methods are shown in Fig 22. The solid line represents the target trajectory, the dash-dotted line represents the trajectory of the proposed method, and the dashed line represents the trajectory under ET-Fuzzy-MPC control. The figure indicates that the proposed method closely aligned with the target trajectory throughout the tracking process, demonstrating excellent performance. In contrast, the ET-Fuzzy-MPC method exhibited a significant lag during the dynamic adjustment process in the initial turning phase. These results can be attributed to the hyperbolic tangent function used in the HLOS guidance strategy in the proposed method, which made timely adjustments to the autonomous vehicle's heading angle and speed, ensuring that the vehicle remained closely aligned with the target trajectory. However, the ET-Fuzzy-MPC method exhibited a slower dynamic response when the target trajectory changed significantly, causing its tracking trajectory to deviate from the target. The proposed method uses an ESO to compensate for odometry errors in real time, significantly reducing the impact of cumulative errors.

**Fig 23:**
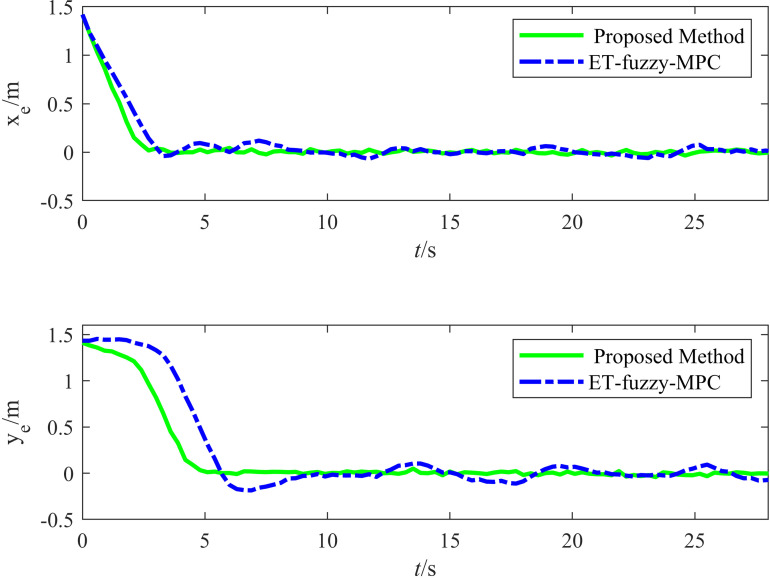
Trajectory deviation in the straight-line driving experiment.

Fig 23 illustrates the variation in the tracking errors in the longitudinal and lateral directions over time during the straight-line driving experiment. The figure clearly illustrates the differences between the performances of the proposed and ET-Fuzzy-MPC methods, as demonstrated by the fact that the proposed method exhibited significant dynamic response capability and steady-state control accuracy in both the longitudinal and lateral directions. The longitudinal direction tracking error curve indicates that the proposed method quickly converged to approximately zero in the initial stage and maintained a minimal fluctuation range throughout the tracking process, demonstrating that its dynamic adjustment capability and steady-state control accuracy were superior to those of the ET-Fuzzy-MPC method. In contrast, the ET-Fuzzy-MPC method exhibited more substantial lateral errors in the initial stage, exhibiting a significantly slower convergence rate and higher steady-state errors than those of the proposed method. This indicates that the ET-Fuzzy-MPC could not quickly adjust the lateral position of the vehicle. In the lateral direction, the error of the proposed method converged rapidly and maintained stable control during dynamic changes in the target trajectory, exhibiting significantly smaller error fluctuations than those of the ET-Fuzzy-MPC method. In contrast, the lateral direction error curve of the ET-Fuzzy-MPC method exhibited greater volatility, especially during the initial adjustment phase, with significantly larger error amplitudes, indicating that the ET-Fuzzy-MPC method possessed a limited capability for making longitudinal adjustments to dynamic targets, hindering high-precision tracking.

Table 8 presents the RMSE and maximum error for $e_{x}$, $e_{y}$, and $e_{\theta }$ for the target-tracking tasks using different control methods. The comparison results between the proposed and ET-Fuzzy-MPC methods are as follows. Regarding the RMSE, the proposed method demonstrated a higher tracking accuracy. For the longitudinal error, the RMSE of the proposed method was 0.0168 m, which was 56.70% lower than that achieved by ET-Fuzzy-MPC (0.0388 m), indicating that the proposed method more accurately followed the target trajectory in the longitudinal direction. For the lateral error, the RMSE of the proposed method was 0.0176 m, which was 68.57% lower than that achieved by ET-Fuzzy-MPC (0.0560 m), indicating that the proposed method exhibited better overall control performance in the lateral direction. In terms of average positional tracking error, the RMS error of the proposed method is 0.0243 m, compared to 0.0681 m for the ET-Fuzzy-MPC method, with a position error reduction rate of 64.29%, further validating the significant advantage of the proposed method in suppressing overall positional errors. For the heading error, the value decreased from 0.1080 rad to 0.0131 rad (a reduction of 87.87%), further validating the superior directional control capability of the proposed method during dynamic heading adjustments. In terms of the maximum error, the proposed method achieved significant reductions in the maximum errors in the longitudinal and lateral directions and heading angle compared to those of the ET-Fuzzy-MPC method, demonstrating stronger robustness and error suppression capability, particularly when handling dynamic target changes. These results indicate that the proposed method achieved high-precision target tracking control in dynamic and complex environments.

**Table 8:** Trajectory deviations in the straight-line driving experiment.

Control Methods	$||e_{x}|{|}_{\text{rms}}$	$||e_{y}|{|}_{\text{rms}}$	$||e_{\theta }|{|}_{\text{rms}}$	$||e_{x}|{|}_{\max}$	$||e_{y}|{|}_{\max}$	$||e_{\theta }|{|}_{\max}$
ET-Fuzzy-MPC	0.0388	0.0560	0.1080	0.1197	0.1124	0.1936
Proposed Method	0.0168	0.0176	0.0131	0.0646	0.0792	0.0353

#### 6.2.4. Experiment 4: Vehicle curved-path driving test.

**Fig 24:**
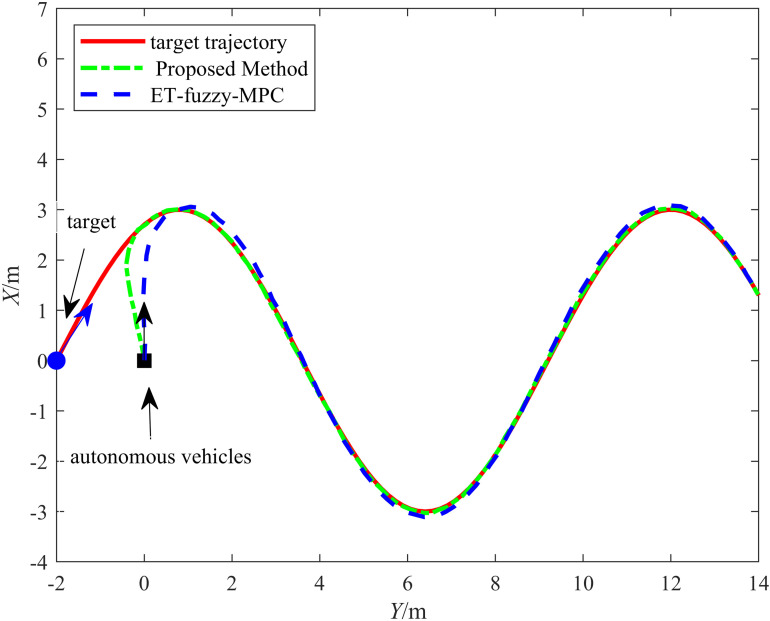
Results of the curved-path driving experiment.

Curved-path driving requires frequent direction changes, increasing the difficulty in achieving adequate target tracking, and it is more susceptible to various factors than straight-line driving. A curved-path driving experiment was conducted to further validate the proposed method's effectiveness. The experimental results of the proposed and MPC methods are presented in Fig 24. The solid line represents the target curve trajectory, the dashed line represents the vehicle trajectory of the ET-Fuzzy-MPC method, and the dash-dotted line represents the vehicle trajectory of the proposed method. As illustrated in Fig 24, the ET-Fuzzy-MPC method exhibited significant deviations in the initial stage and when the target trajectory was highly curved. In contrast, the proposed method tracked the target quickly and stably throughout the tracking process. According to the experimental results for the two methods, the proposed algorithm more effectively suppressed disturbances while tracking the target.

**Fig 25:**
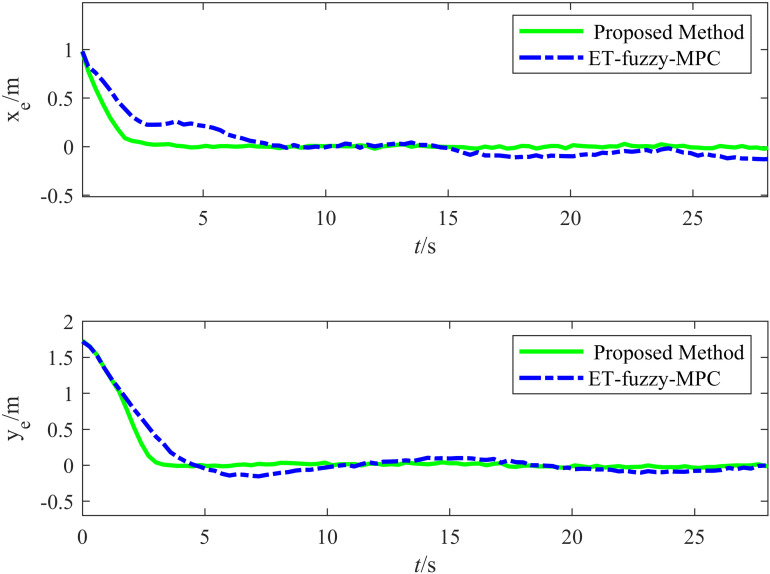
Trajectory deviations in the curved-path driving experiment.

Fig 25 presents the variations in the tracking errors in the longitudinal and lateral directions over time during the curved-path driving experiment. The figure indicates that the proposed method exhibited significant dynamic response capability and steady-state control accuracy in the longitudinal and lateral directions. For the tracking error in the longitudinal direction, the proposed method quickly converged in the initial stage, and after stabilizing, the error approached zero. In contrast, the ET-Fuzzy-MPC method exhibited a longer adjustment time while converging the errors, and its steady-state error was significantly higher than that of the proposed method. In the lateral direction, the error of the proposed method converged rapidly and remained stable, even after the target path changed. However, the ET-Fuzzy-MPC method exhibited greater fluctuation amplitudes while it adjusted the longitudinal error. These results demonstrate that the proposed method exhibited high robustness and practicality for target-tracking tasks involving complex target paths.

Table 9 presents the RMSE and maximum errors for $e_{x}$, $e_{y}$, and $e_{\theta }$ for the curved-path driving target-tracking task using both control methods. The proposed method demonstrated a significant advantage regarding the longitudinal error, with an RMSE of 0.0206 m, which was lower than that of the ET-Fuzzy-MPC method (0.0745 m) by approximately 72.35%. In the lateral direction, the proposed method achieved an RMSE of 0.0297 m, approximately 52.86% lower than that of the ET-Fuzzy-MPC method (0.0630 m). Furthermore, the proposed method reduces the average positional tracking error from 0.0976 m of the ET-Fuzzy-MPC method to 0.0361 m, a reduction of 62.95%. These results indicate that the proposed method effectively compensated for the cumulative errors using the ESO and quickly adjusted the dynamic errors using the HLOS strategy, maintaining a higher tracking accuracy for complex path changes. For the heading error, the RMSE of the proposed method was 0.0149 rad, which was lower than that of the ET-Fuzzy-MPC method (0.0290 rad) by 48.62%. This demonstrates the proposed method's ability to accurately adjust the vehicle's direction under complex path conditions. The maximum longitudinal error of the proposed method was 0.1185 m, which was 21.68% lower than that of the ET-Fuzzy-MPC method (0.1513 m). In contrast, the maximum lateral error of the proposed method was 0.1015 m, which was 29.51% lower than that of the ET-Fuzzy-MPC method (0.1440 m), and the maximum position error of this method was 0.1560 m. These results validate the ability of the proposed method to suppress positional deviations under dynamic path changes, which allowed the vehicle to accurately follow the target path and avoid excessive deviations caused by dynamic disturbances or path changes. In addition, the maximum heading error of the proposed method was 0.0511 rad, which was 29.71% lower than that of the ET-Fuzzy-MPC method (0.0727 rad), further validating the efficiency and stability of the proposed method for directional control during dynamic path adjustments.

**Table 9:** Trajectory deviations in the curved-path driving experiment.

Control Methods	$||e_{x}|{|}_{\text{rms}}$	$||e_{y}|{|}_{\text{rms}}$	$||e_{\theta }|{|}_{\text{rms}}$	$||e_{x}|{|}_{\max}$	$||e_{y}|{|}_{\max}$	$||e_{\theta }|{|}_{\max}$
ET-Fuzzy-MPC	0.0745	0.0630	0.0290	0.1513	0.1440	0.0727
Proposed Method	0.0206	0.0297	0.0149	0.1185	0.1015	0.0511

#### 6.2.5. Experiment 5: Vehicle sharp turn driving test.

**Fig 26:**
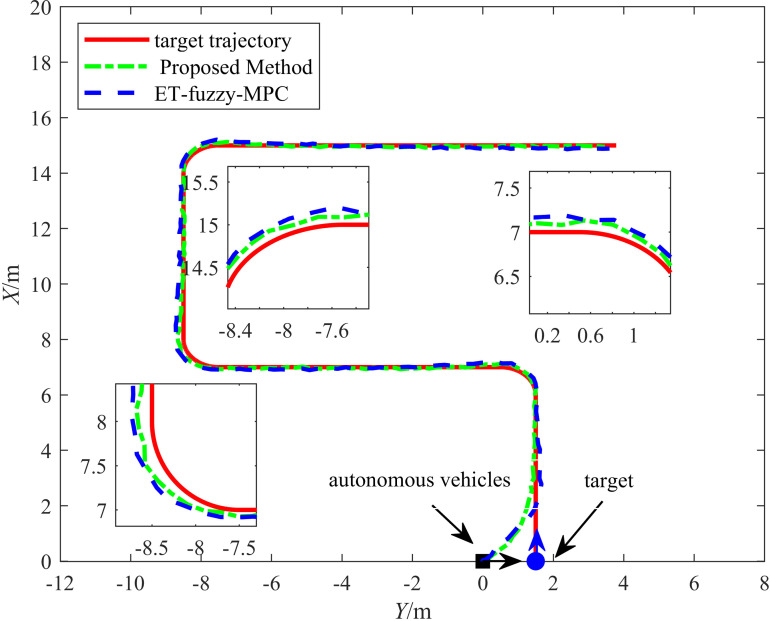
Sharp turn driving experiment.

An additional high-speed sharp turn target tracking experiment was conducted to further validate the adaptability of the proposed method in challenging environments. We introduced a high-difficulty scenario where the vehicle operated at 2.0 m/s, which is the maximum achievable speed of the experimental platform. Fig 26 illustrates the trajectory of the sharp turn driving experiment. The detailed view reveals that the proposed method achieves more accurate target tracking during the sharp turn phase than the MPC method from [16]. The trajectory based on ET-Fuzzy-MPC deviates considerably from the target owing to its slower adaptation to rapid heading changes, whereas the proposed method ensures smooth and accurate target tracking throughout the driving process.

**Fig 27:**
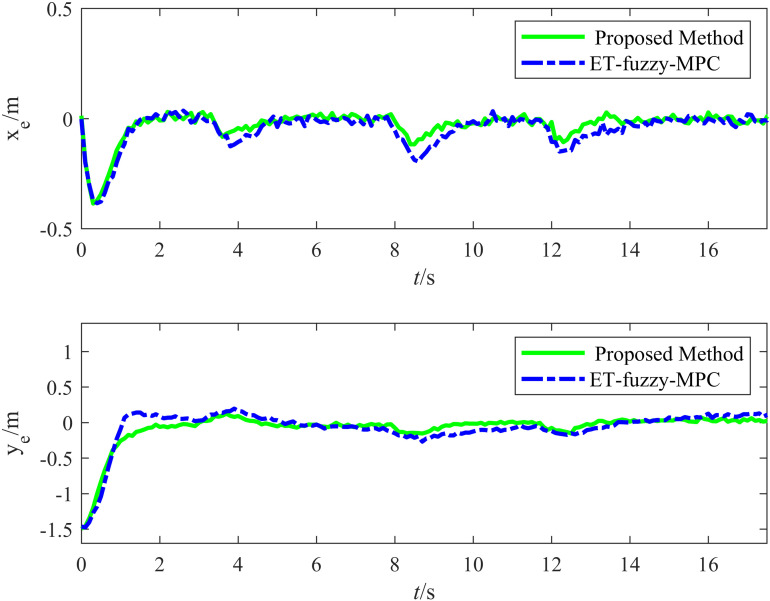
Sharp turn driving experiment.

Fig 27 illustrates the variation of longitudinal and lateral errors over time in the sharp turn driving experiment. Compared to ET-Fuzzy-MPC, the proposed method maintains a lesser tracking error. The longitudinal tracking error graph indicates that both methods maintain relatively low errors on straight paths. Conversely, ET-Fuzzy-MPC exhibits greater error fluctuations at sharp turns compared to the proposed method. The proposed method also demonstrates a faster error convergence rate, highlighting its superiority in handling sudden turns.

Table 10 presents the RMSE and maximum error for $e_{x}$, $e_{y}$, and $e_{\theta }$ in the sharp turn tracking task using different control methods. For RMSE, the proposed method achieves a longitudinal RMSE of 0.0337 m, which is 41.99% lower than the that of the ET-Fuzzy-MPC method (0.0581 m). In terms of lateral error, the proposed method's RMSE is 0.0586 m, i.e., a reduction of 46.39% compared to that for the ET-Fuzzy-MPC method (0.1093 m). For overall position error, the proposed method achieves an RMS error of 0.0676 m, i.e., a 45.39% reduction compared to the overall error of the ET-Fuzzy-MPC method (0.1238 m), further validating the proposed method's significant advantage in suppressing overall position errors. Regarding maximum error, the proposed method demonstrates superior error suppression in all directions. The maximum longitudinal error is reduced by 39.92% from 0.1939 m for the ET-Fuzzy-MPC method to 0.1165 m. The maximum lateral error decreases by 43.02% from 0.2680 m for the ET-Fuzzy-MPC method to 0.1527 m. Additionally, the maximum position error of the proposed method is 0.192 m. These results indicate that the proposed method significantly outperforms the ET-Fuzzy-MPC method in trajectory tracking during sharp turns, particularly in terms of error suppression and trajectory stability, providing an effective solution for high-precision tracking control in complex paths.

**Table 10:** Error evaluation metrics for sharp turn driving experiment.

Control Methods	$||e_{x}|{|}_{\text{rms}}$	$||e_{y}|{|}_{\text{rms}}$	$||e_{\theta }|{|}_{\text{rms}}$	$||e_{x}|{|}_{\max}$	$||e_{y}|{|}_{\max}$	$||e_{\theta }|{|}_{\max}$
ET-Fuzzy-MPC	0.0581	0.1093	0.0681	0.1939	0.2680	0.2072
Proposed Method	0.0337	0.0586	0.0586	0.1165	0.1527	0.1852

## 7. Discussion

The proposed HLOS strategy exhibits significant advantages compared to the traditional LOS strategy. The traditional LOS strategy maintains a fixed guidance law under various operating conditions, leading to considerable tracking errors when the target path has curvature variations. In contrast, the HLOS strategy uses a nonlinear adjustment mechanism that dynamically modifies the desired heading angle and velocity based on real-time tracking errors, thereby enhancing the dynamic response and tracking accuracy of the autonomous vehicle. Moreover, compared to the traditional PID or MPC control methods, ADRC does not require precise modeling and can leverage ESO to estimate total system disturbances in real time, providing superior robustness. Additionally, this study utilizes wheel odometry as the primary source of feedback. Compared to vision-based or LiDAR-based localization methods, wheel odometry offers advantages such as computational simplicity, strong real-time performance, and high resistance to environmental disturbances. However, wheel odometry is susceptible to cumulative errors over extended periods, especially on rough terrain or long-distance travel, where errors continuously accumulate and affect tracking accuracy. This study used ESO to estimate and compensate for odometry errors, ensuring high tracking accuracy over short durations and in relatively flat environments. However, the effectiveness of this method may degrade in scenarios with drastic terrain variations, such as rugged roads or unstructured environments. Therefore, future research could consider integrating external sensor information from vision-based SLAM and LiDAR with wheel odometry to mitigate the impact of severe terrain variations on tracking accuracy. This study primarily focused on the tracking accuracy of autonomous vehicles but did not consider their obstacle-avoidance capabilities. In complex environments, vehicles may encounter dynamic or static obstacles, and the absence of an obstacle avoidance mechanism could lead to collisions or tracking failures. Future research will focus on integrating path-planning algorithms with environmental perception technologies to enable dynamic obstacle avoidance while incorporating intelligent decision-making methods for trajectory optimization. This will enhance the autonomy and safety of the system, ensuring the stable operation of autonomous vehicles in complex environments.

## 8. Conclusions

This study addressed the target-tracking challenges faced by autonomous vehicles in complex environments by proposing a control method that combines a hyperbolic tangent LOS guidance strategy and odometry compensation based on an ESO. The main conclusions of this study are as follows.

(1) The hyperbolic tangent LOS guidance strategy significantly improved the vehicle's dynamic response capabilities for tracking along complex paths. This strategy excelled during abrupt path changes, effectively reducing path deviations.
(2) Using the ESO for real-time observation and compensation of the system disturbances successfully reduced the negative impact of the odometry errors on the overall system positioning accuracy, enhancing the robustness of the system during long-term operations.
(3) Both simulations and real-world experiments validated the effectiveness of the proposed method, which demonstrated superior tracking performance compared to the MPC control strategy in dynamic environments, particularly in terms of target tracking accuracy and system stability.

## Supporting information

S1 File Research data. (XLSX)
